# Continuing medical education in renal pathology: current practices and needs among nephrologists

**DOI:** 10.1186/s12909-026-08798-4

**Published:** 2026-02-12

**Authors:** Qianqian Han, Jiamin Yuan, Lijun Zhao, Fang Liu

**Affiliations:** 1 Department of Pathology, West China Hospital of Sichuan University, Chengdu, China; 2 Department of Nephrology, Laboratory of Diabetic Kidney Disease, Kidney Research Institute, West China Hospital of Sichuan University, Guoxuexiang 37, Chengdu, 610041 Sichuan China; 3 Department of General Practice Ward, West China Hospital, International Medical Center, General Practice Medical Center, Sichuan University, Ward, Chengdu, China

**Keywords:** Continuing medical education (CME), Renal pathology, Nephrologists

## Abstract

**Objective:**

To investigate nephrologists’ attitudes toward renal pathology continuing medical education (CME), current problems, as well as their demands for CME

**Methods:**

A self-designed questionnaire survey themed “The Value of Renal Pathology in Clinical Diagnosis, Treatment and CME Across Medical Institutions at All Levels” was conducted among 281 nephrologists. Descriptive statistical analysis, chi-square tests and logistic regression analysis were performed on the survey data.

**Results:**

A total of 256 valid questionnaires were included, with the demographic profile as follows: 56.6% females, 29.7% aged over 40 years, 26.6% with senior titles or above, 41% with over 10 years of working experience, and 42.2% working in Class B tertiary hospitals and below. All respondents recognized the clinical value of renal pathology, with female and senior physicians showing more positive attitudes. Physicians in primary institutions faced prominent difficulties in linking pathological findings to clinical manifestations. Only 53.1% of the respondents participated in at least one training session per year, mainly due to time conflicts and lack of targeted content. Most physicians regarded clinico-pathological case analysis as the core training content, while senior/title-holding physicians also needed training on renal pathology advances and new technologies. And primary institutions demanded support for specimen referral and telepathology consultation.

**Conclusion:**

Renal pathology training for nephrologists urgently needs to be strengthened and expanded. The content should cover clinico-pathological case analysis, pathological report interpretation, research advances and new technologies, according to the needs-oriented principle. Besides, practical remote slide-reading training was suggested.

**Supplementary Information:**

The online version contains supplementary material available at 10.1186/s12909-026-08798-4.

## Introduction

Renal diseases represent a major threat to human health. Not only do they maintain a persistently high incidence rate, but their pathological types also directly determine the selection of treatment regimens and patient’s prognosis. For instance, the Oxford classification of IgA nephropathy guides prognostic assessment, while the ISN/RPS classification of lupus nephritis directly dictates the strategy for selecting immunosuppressants [1]. However, the pathological types of such diseases are complex and diverse. Additionally, there is often a significant disconnect between patients’ clinical manifestations and their actual pathological changes, making it difficult to accurately infer pathological types based solely on symptoms. As a result, renal diseases remain the key focus and major challenge in clinical diagnosis and treatment [2]. For example, the nodular lesions of diabetic nephropathy, which frequently require differentiation from membranoproliferative glomerulonephritis, renal amyloidosis, and fibrillary glomerulopathy [1, 3]. Precise differentiation cannot be accomplished based solely on clinical manifestations and laboratory examinations, renal pathological diagnosis was necessary, including immunofluorescence and electron microscopy [3]. Therefore, renal pathology serves as the gold standard for the diagnosis and treatment of renal diseases, and the ability to interpret renal pathological findings constitutes a critical component of the core clinical competencies for nephrologists.

With the further advancement of the hierarchical diagnosis and treatment policy in China, nephrologists across medical institutions at all levels have developed differentiated clinical roles: tertiary hospitals focus on managing complex cases and applying new technologies; secondary hospitals specialize in the accurate diagnosis and treatment of common diseases; and primary medical institutions take responsibility for the early screening of chronic kidney disease and decision-making regarding patient referrals [4]. This division of labor has resulted in substantial disparities in the understanding of renal pathology, clinical demand for renal pathology services, and heterogeneity of pathological reports among physicians at different levels. Furthermore, many medical institutions are confronted with widespread challenges, including a shortage of specialized renal pathologists and limited accessibility to renal pathological techniques and resources [5]. Meanwhile, technological innovations in the field of renal pathology are rapidly reshaping clinical practice—molecular pathology techniques provide a novel basis for predicting the efficacy of immunosuppressants; digital pathology and teleconsultation systems have broken down geographical barriers to resource access; and artificial intelligence-assisted diagnosis has greatly improved the efficiency of pathological analysis [6, 7]. These advances have placed higher demands on the speed of physicians’ knowledge updating. The traditional “one-time learning” model in medical education is no longer able to meet clinical needs, necessitating continuing medical education (CME) to achieve knowledge iteration.

Current domestic research on renal pathology education mostly focuses on the innovation of teaching methods, such as visual-spatial dimension integration in pathology learning, 3D pathology animations, whole-slide imaging technology, and the construction of a multidisciplinary team-based teaching model combining problem-based learning and case-based learning with multimodal approaches [8–11]. Nevertheless, the subjects of these studies are mostly undergraduate students, interns, or resident physicians. In fact, with the rapid development of diagnostic and therapeutic technologies and the continuous evolution of disease spectra, the necessity of CME in renal pathology has become increasingly prominent for practicing physicians at all levels. To date, no targeted survey research has been performed to explore clinical physicians’ perceptions of the value of renal pathology, their demands for CME, and the pertinent existing challenges in this field.

Against this backdrop, guided by the needs of nephrologists, this study intends to conduct a questionnaire survey among nephrologists working in medical institutions at all levels. This research will focus on the following aspects: First, the importance of the practical application of renal pathology in the diagnosis and management of kidney diseases. Second, main difficulties of participation in renal pathology CME of nephrologists across medical institutions of different levels. Third, specific implementation methods and improvement suggestions for CME in renal pathology. Fourth, current status and future perspectives on artificial intelligence (AI) pathology and third-party pathology services. These findings of this study will provide empirical evidence for the development of CME in renal pathology.

## Subjects and methods

### Study subjects

From July to August 2025, a convenience sampling method was adopted to recruit physicians who have obtained medical practitioner qualifications and are engaged in nephrology-related work across China as study subjects, including physicians who have obtained or are undergoing nephrology specialist training, internists managing kidney disease patients, and formally trained dialysis physicians. The participants covered all age groups, all physician career stages, and medical institutions ranging from primary care facilities to Grade III Class A hospitals. The sample size of this study was estimated using Cochran [12]. Core parameters were set based on conventions from similar studies: expected prevalence (p) = 0.5, margin of error (d) = 0.06, and Z-value = 1.96 corresponding to a 95% confidence level. Substituting these parameters into the formula n_0_ = Z^2^×p×(1 − p)/d^2^, the minimum theoretical sample size was calculated to be 267. After adjusting for a 15% non-response rate, the required sample size was 315. A total of 281 questionnaires were actually retrieved in this study with response rate of 89.2%, and 256 valid questionnaires were included for analysis with response rate of 91.1% (Supplemental Fig. 1). The high response rate and valid rate can ensure the representativeness of the sample and meet the research requirements.

### Study methods

This study adopted a cross-sectional survey design, which was conducted in accordance with the ethical principles of the Declaration of Helsinki, and was approved by the Scientific Ethics Committee of West China Hospital, Sichuan University. All participants provided informed consent for their participation in this study. The questionnaire was developed through an extensive review of domestic and international literature and expert consultations to ensure its scientific rigor. The questionnaire consisted of 36 questions in total, focusing on four core aspects: (1) Basic demographic information of respondents, including age, sex, professional title, years of clinical practice, and the tier of the affiliated medical institution; (2) Current problems and difficulties related to renal pathology in clinical practice, including the challenges in renal pathology interpretation, the current status and issues of renal pathological diagnosis in primary care settings, the application of and concerns about AI in renal pathological diagnosis, third-party pathological testing institutions, and clinical-pathological communication; (3) Current status of renal pathology-related training and the demands and directions for future CME; (4) Clinician’ evaluation of the significance of renal pathology in clinical practice and disease diagnosis.

The questionnaire included closed-ended questions, multiple-choice questions, and open-ended questions. In addition, the design of the questionnaire incorporated the opinions of experts in nephrology, renal pathology, and medical education to ensure the validity and relevance of its content. Content validity was further confirmed through two rounds of expert reviews by 10 interdisciplinary specialists, with all items achieving an item-level content validity index (I-CVI) ≥ 0.78 and an overall scale-level content validity index (S-CVI) of 0.92. Items with low validity or substantial expert feedback were refined to enhance their clarity and relevance based on the comments received. Prior to the formal survey, a pilot test was conducted among 10 nephrologists. The final version of the questionnaire was determined after thorough discussion and revision.

The questionnaire was distributed via WeChat groups relevant to nephrologists. Physicians voluntarily completed the questionnaire anonymously and submitted it online, with each IP address restricted to one submission only. After the questionnaires were collected by investigators, the data were collated by one researcher and verified for accuracy by another before being aggregated. Questionnaires with random or obviously invalid responses were excluded based on criteria such as response time and quality control questions. Questionnaires from respondents in departments unrelated to nephrology were also discarded. The Cronbach’s alpha coefficient of the questionnaire was 0.93, indicating good internal consistency reliability.

### Statistical analysis

Statistical analysis was performed using IBM SPSS 27.0. Count data were expressed as frequencies and percentages [n (%)], and analyzed using descriptive statistics and the chi-square (χ²) test. Univariate and multivariate logistic regression analyses, adjusted for sex, age, working years, professional title, and hospital grade as necessary, were performed to assess differences in response indicators among groups. A dichotomous analysis was applied to all items in this study. Some were directly dichotomized based on option selection status, while for Likert scale items, “Extremely Important” responses were categorized as a separate group, with all remaining responses combined into another. *P* < 0.05 was considered statistically significant.

## Results

### Basic characteristics of participating nephrologists

Among the 256 participating nephrologists, 145 (56.6%) were female and 111 (43.4%) were male. A total of 180 (70.3%) were aged ≤ 40 years, while 76 (29.7%) were aged > 40 years. In terms of clinical practice duration, 151 (59.0%) had less than 10 years of experience, and 105 (41.0%) had more than 10 years of experience, among whom 34 (13.7%) had over 20 years of experience. Regarding professional titles, 188 (73.4%) held titles at or below the level of Attending Physician, and 68 (26.6%) held titles at or above the level of Associate Chief Physician. As for the affiliated institutions, 148 (57.8%) worked in Grade III Class A hospitals, and 108 (42.2%) worked in hospitals at or below the level of Grade III Class B. In terms of departmental affiliation, 145 (56.6%) worked in nephrology wards, and 111 (43.4%) worked in other departments including nephrology outpatient clinics, hemodialysis/peritoneal dialysis centers, renal pathology departments, and kidney transplantation centers (Supplemental Tables 1–6).

### Importance of renal pathology in the clinical practice of nephrologists

**Fig. 1:**
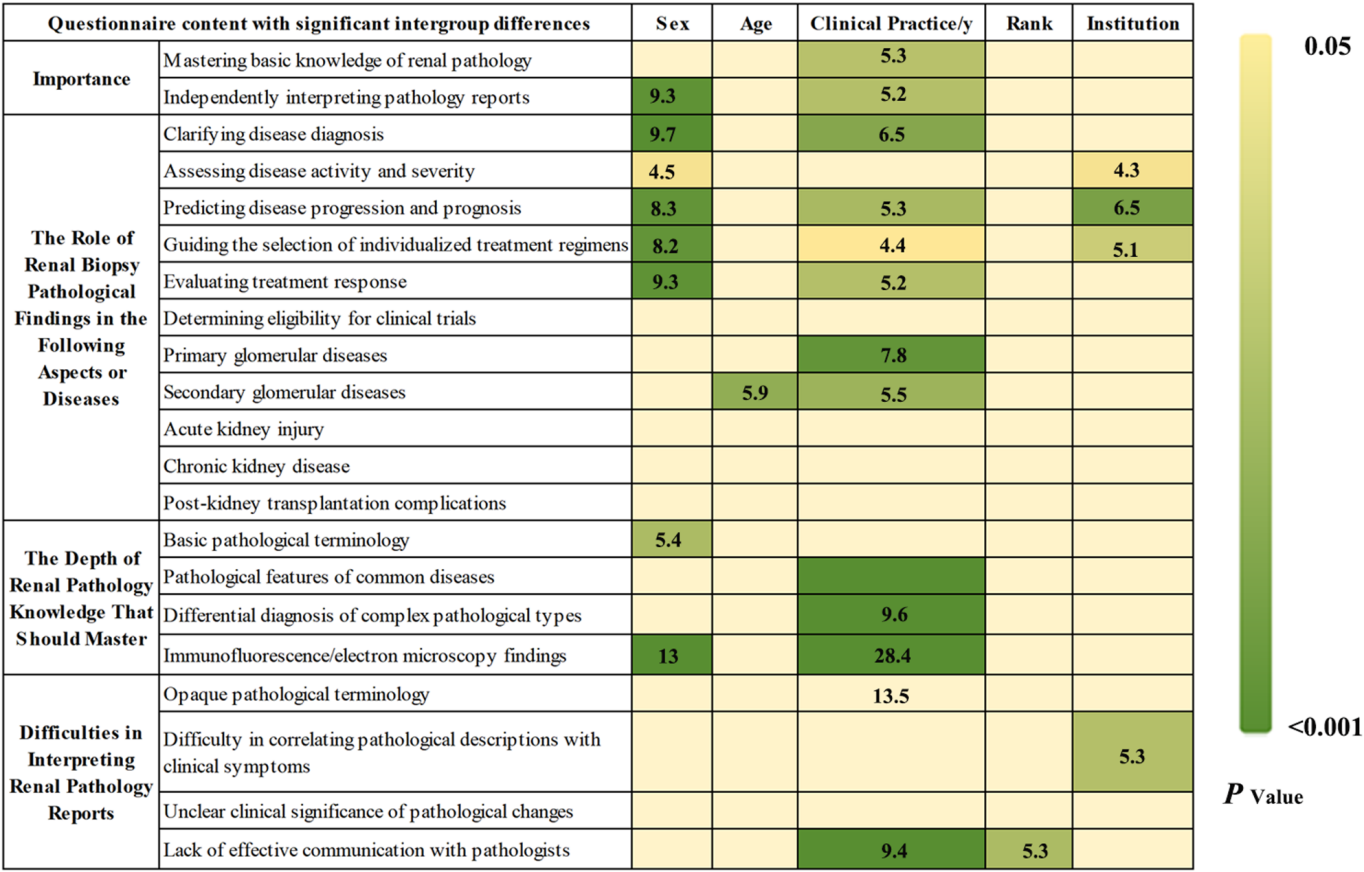
Analysis of the importance of renal pathology in the clinical practice of nephrologists and the current existing problems

A total of 193 physicians (75.4%) considered mastering basic renal pathology knowledge to be extremely important, and 180 physicians (70.3%) held the same view regarding the ability to independently interpret core information of renal biopsy reports. Compared with males, female physicians demonstrated a more positive attitude toward “Ability to Independently Interpret Pathology Reports” (*p* = 0.001, OR = 2.62, 95% CI = 1.48–4.64). More than 70% of the physicians agreed that renal pathology was extremely important for clarifying disease diagnosis, assessing disease activity and severity, predicting disease progression and prognosis, guiding the selection of individualized treatment regimens, and evaluating treatment response. Compared with males, those female physicians showed a significantly more favorable attitude toward the role of renal biopsy pathology in the importance of disease diagnosis (*p* = 0.001, OR = 2.91, 95% CI = 1.54–5.48), progression prediction (*p* = 0.002, OR = 2.53, 95% CI = 1.41–4.55), treatment section (*p* = 0.003, OR = 2.46, 95% CI = 1.37–4.43), as well as determining eligibility for clinical trials (*p* = 0.04, OR = 1.73, 95% CI = 1.02–2.92) (Fig. 1 & Supplemental Tables 1 & 6, *P* < 0.05).

On the one hand, 208 physicians (81.3%) believed that basic pathological terminology should be mastered, and 231 physicians (90.2%) emphasized the necessity of grasping the pathological features of common diseases. In addition, more than 60% of the physicians agreed that the differential diagnosis of complex pathological types and the clinical significance of immunofluorescence/electron microscopy results were also essential learning content. Compared with male physicians, female physicians showed a more positive attitude toward mastering basic pathological terminology (*p* = 0.014, OR = 2.26, 95% CI = 1.18–4.34) and interpreting immunofluorescence or electron microscopy results (*p* < 0.001, OR = 3.24, 95% CI = 1.83–5.75). Similarly, physicians with over 10 years of clinical experience were more inclined to master the pathological features of common diseases (*p* = 0.001, OR = 22.21, 95% CI = 3.87-127.49), the differential diagnosis of complex pathological types (*p* = 0.002, OR = 3.72, 95% CI = 1.65–8.41), and the interpretation of immunofluorescence or electron microscopy results *p* < 0.001, OR = 4.67, 95% CI = 2.06–10.65) than those with less than 10 years of experience (Fig. 1 & Supplemental Tables 1 & 6).

On the other hand, more than 70% of the physicians identified the main difficulties in interpreting pathology reports as the inability to correlate pathological descriptions with clinical symptoms and the unclear clinical significance of different pathological changes. This issue was particularly prominent among physicians working in hospitals at or below the level of Grade III Class B (85 cases, 78.7%) compared with those in Grade III Class A hospitals (97 cases, 65.5%). In addition, approximately 50% of the physicians reported the lack of effective communication with pathologists were major obstacles, and this sentiment was more frequently expressed by physicians with over 10 years of clinical experience (*p* = 0.011, OR = 2.47, 95% CI = 1.23–4.96). Collectively, these findings highlight the critical importance of CME in renal pathology for nephrologists (Fig. 1 & Supplemental Tables 1& 6).

### Current Status, Problems, and challenges of continuing medical education in renal pathology for nephrologists

Regarding the channels for learning and updating renal pathology knowledge, attending academic conferences/lectures/training courses was the most commonly adopted approach (196 physicians, 76.6%). In addition, a large number of physicians chose to read professional books and journal articles (159 physicians, 62.1%) and utilize online learning resources such as online courses, databases, and pathological atlases (156 physicians, 60.9%). Furthermore, some physicians acquired knowledge through medical school courses, resident/specialist standardized training, communication with pathologists, and case conferences.

**Fig. 2:**
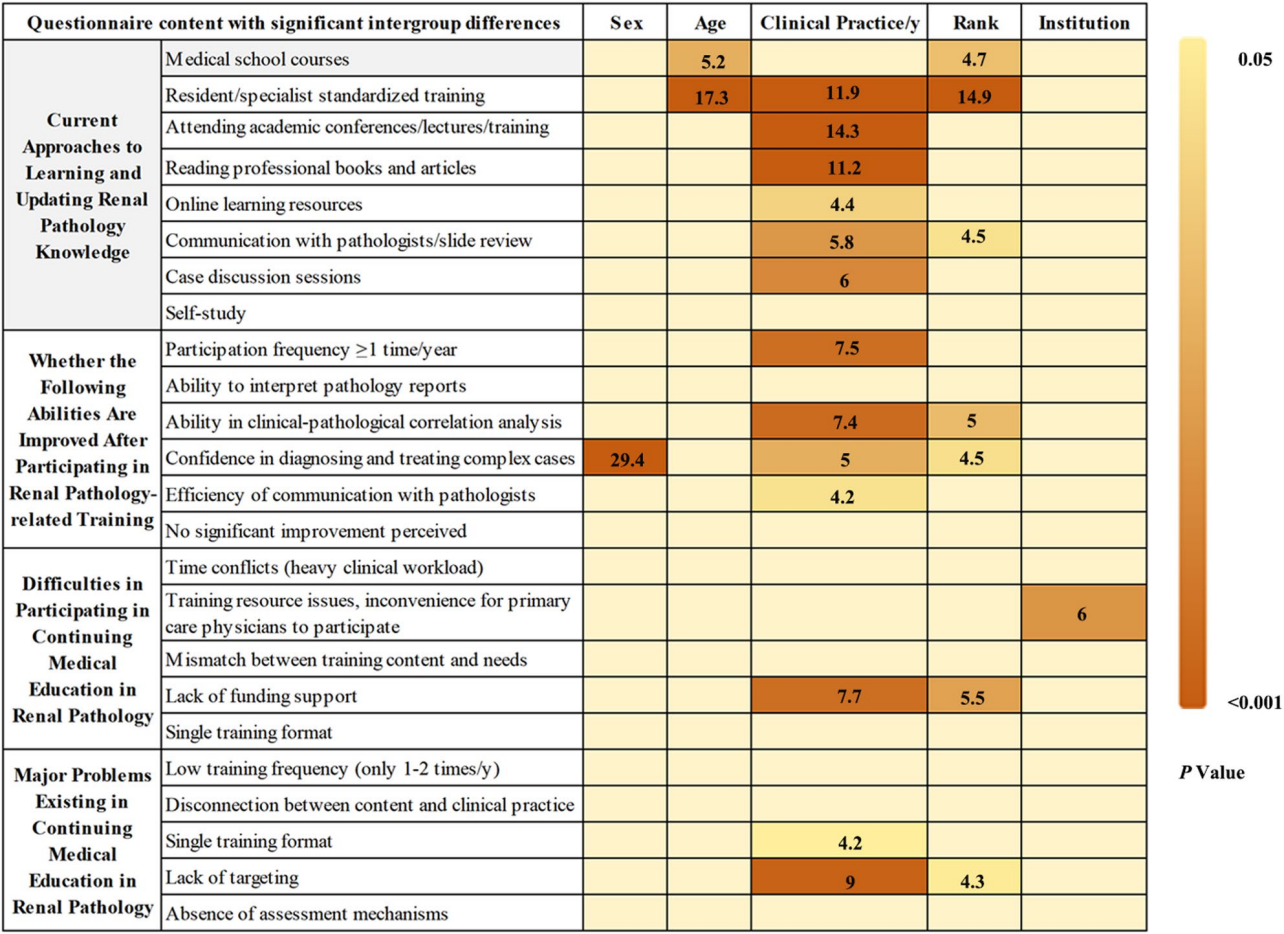
Current status, problems and difficulties of nephrologists’ participation in renal pathology-related training

Physicians aged ≤ 40 years were more likely to choose standardized physician training (65.0% vs. 36.8%) (*p* = 0.046, OR = 0.45, 95% CI = 0.21–0.99), compared with those aged over 40 years. In contrast, compared with those with less than 10 years of experience, physicians with over 10 years of clinical experience were more inclined to update their pathological knowledge through academic conferences (88.6% vs. 68.2%) (*p* = 0.001, OR = 5.41, 95% CI = 1.95–14.96), professional books and journal articles (74.3% vs. 53.6%) (*p* < 0.001, OR = 4.25, 95% CI = 1.88–9.60), or online learning resources (68.6% vs. 55.6%) (*p* = 0.011, OR = 2.76, 95% CI = 1.27–6.01) (Fig. 2 & Supplemental Tables 2 & 6).

Currently, only 136 physicians (53.1%) participated in renal pathology-related training at least once a year, including academic conferences and pathological slide review sessions. These participants reported that such training significantly improved their ability in clinical-pathological correlation analysis, the accuracy of pathology report interpretation, and their confidence in diagnosing and treating complex cases, especially among physicians with over 10 years of clinical experience and those holding titles at or above the level of Associate Chief Physician.

At present, the main difficulties in participating in CME in renal pathology include time conflicts, the concentration of training resources in large cities (which hinders the participation of physicians from primary institutions), the mismatch between training content and personal needs, insufficient funding support, and the single training format. And physicians with over 10 years of clinical experience were more likely to regard insufficient funding support, such as training fees and travel expenses, as a major barrier (*p* = 0.012, OR = 2.51, 95% CI = 1.23–5.11). In contrast, physicians working in hospitals at or below the level of Grade III Class B were more likely to identify the concentration of training resources in large cities and the inconvenience of participation for primary care physicians as the main difficulties (77.8% vs. 63.5% for Grade III Class A hospitals) (*p* = 0.009, OR = 2.21, 95% CI = 1.22–3.98) (Fig. 2 & Supplemental Tables 2 & 6).

Regarding the current training formats, 183 physicians (71.5%) considered low training frequency to be the main problem; 166 physicians (64.8%) pointed out the disconnection between training content and clinical practice, for example overemphasis on pure theory and lack of case analysis); 164 physicians (64.1%) criticized the single training format, for example only offline lectures without online playback; and 142 physicians (55.5%) believed that the lack of targeted training content was the key issue (Fig. 2 & Supplemental Table 2).

### Problems and Solutions in Renal Pathology Practice at Primary Institutions; Value of AI and Third-Party Testing Institutions

**Fig. 3:**
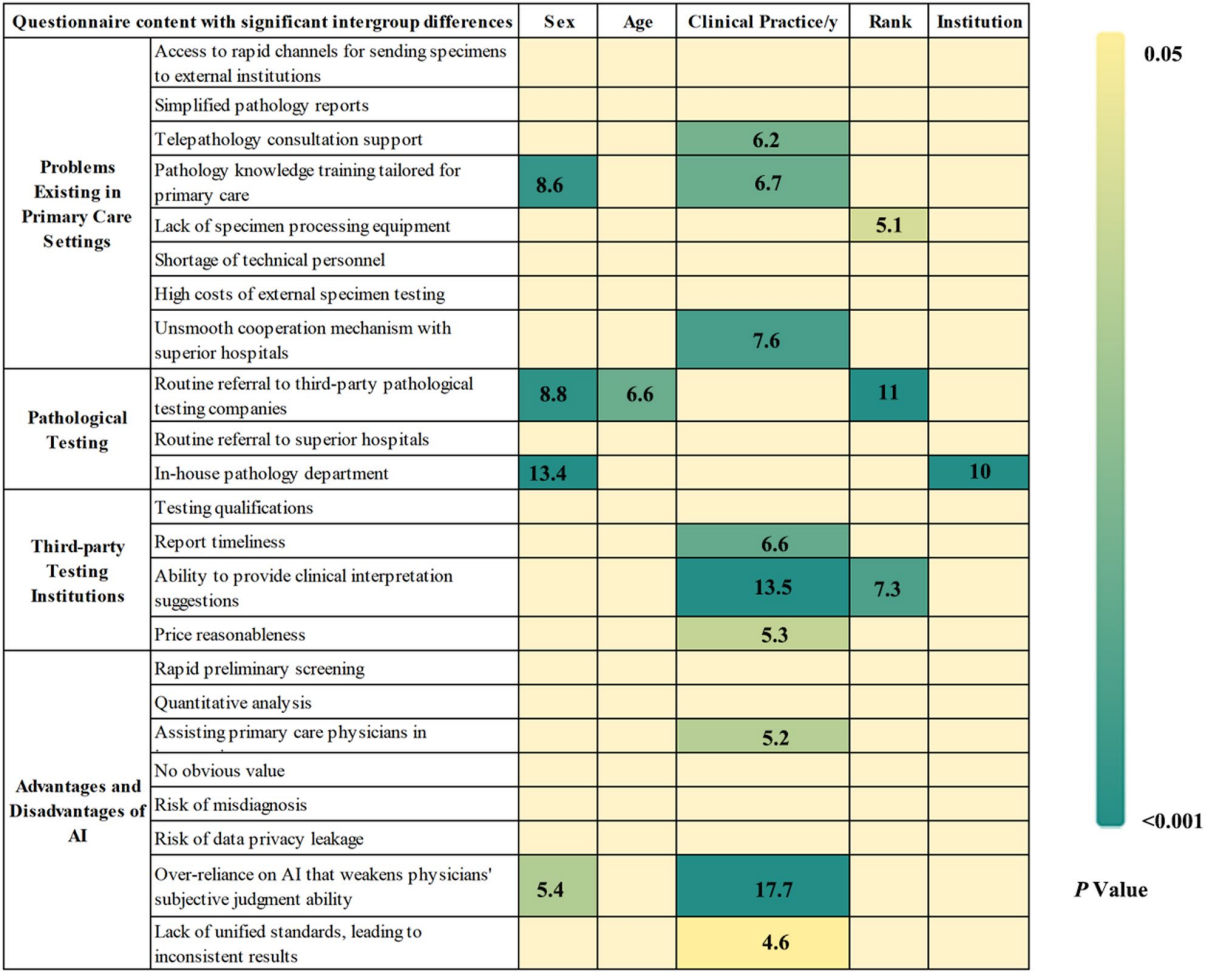
Survey research on primary care renal pathology, pathological artificial intelligence (AI), and third-party testing companies

For primary medical institutions, 196 physicians (76.6%) believed that the core need in renal pathology was access to rapid channels for sending specimens to external institutions, and 186 physicians (72.7%) considered telepathology consultation support to be a core requirement. In addition, 177 physicians (69.1%) and 159 physicians (62.1%) supported pathology knowledge training tailored for primary care settings and simplified pathology reports, respectively. Physicians with over 10 years of clinical experience regarded telepathology consultation support (*p* = 0.013, OR = 2.93, 95% CI = 1.26–6.82) for primary care physicians as core needs, while female physicians showed a more positive attitude toward pathology knowledge training (*p* = 0.001, OR = 2.68, 95% CI = 1.51–4.76) (Fig. 3 & Supplemental Tables 3 & 6).

A total of 209 physicians (81.5%) and 195 physicians (76.2%) identified insufficient technical personnel and the lack of specimen processing equipment as the main restrictive factors for carrying out renal pathology-related work at primary institutions. However, compared with physicians with less than 10 years of clinical experience, those with over 10 years of experience were more likely to consider the unsmooth cooperation mechanism with superior hospitals (*p* = 0.005, OR = 2.82, 95% CI = 1.37–5.81) as a major problem.

A total of 107 nephrologists (41.8%) reported that their institutions routinely sent renal biopsy specimens to third-party pathological testing companies, and 72 nephrologists (28.1%) stated that their institutions regularly sent specimens to superior hospitals for renal pathology testing. These physicians believed that trust in third-party institutions mainly depended on testing qualifications (85.5%), report timeliness (64.1%), and the ability to provide clinical interpretation suggestions (62.9%). In particular, physicians with over 10 years of clinical experience attached greater importance to report timeliness (*p* = 0.003, OR = 3.35, 95% CI = 1.49–7.51), the ability to provide clinical interpretation suggestions (*p* < 0.001, OR = 6.31, 95% CI = 2.50-15.89), and price reasonableness (*p* = 0.007, OR = 2.6, 95% CI = 1.30–5.18) (Fig. 3, Supplemental Tables 3 & 6).

Regarding AI, 88.7% of physicians recognized its potential value in renal pathology. Compared with physicians with less than 10 years of clinical experience, those with over 10 years of experience attached more importance to the role of AI in assisting primary care physicians in interpreting pathology reports (78.1% vs. 64.9%) (*p* = 0.009, OR = 3.05, 95% CI = 1.32–7.05) (Fig. 3, Supplemental Tables 3 & 6). At the same time, physicians also expressed concerns about the application of AI, including the risk of misdiagnosis (especially for rare and complex cases), the leakage of patients’ pathological image data, the lack of unified standards for AI systems, and the potential weakening of physicians’ subjective judgment ability due to over-reliance on AI. These concerns were particularly prominent among physicians with over 10 years of clinical experience.

### Approaches and implementation measures for continuing medical education in renal pathology

**Fig. 4:**
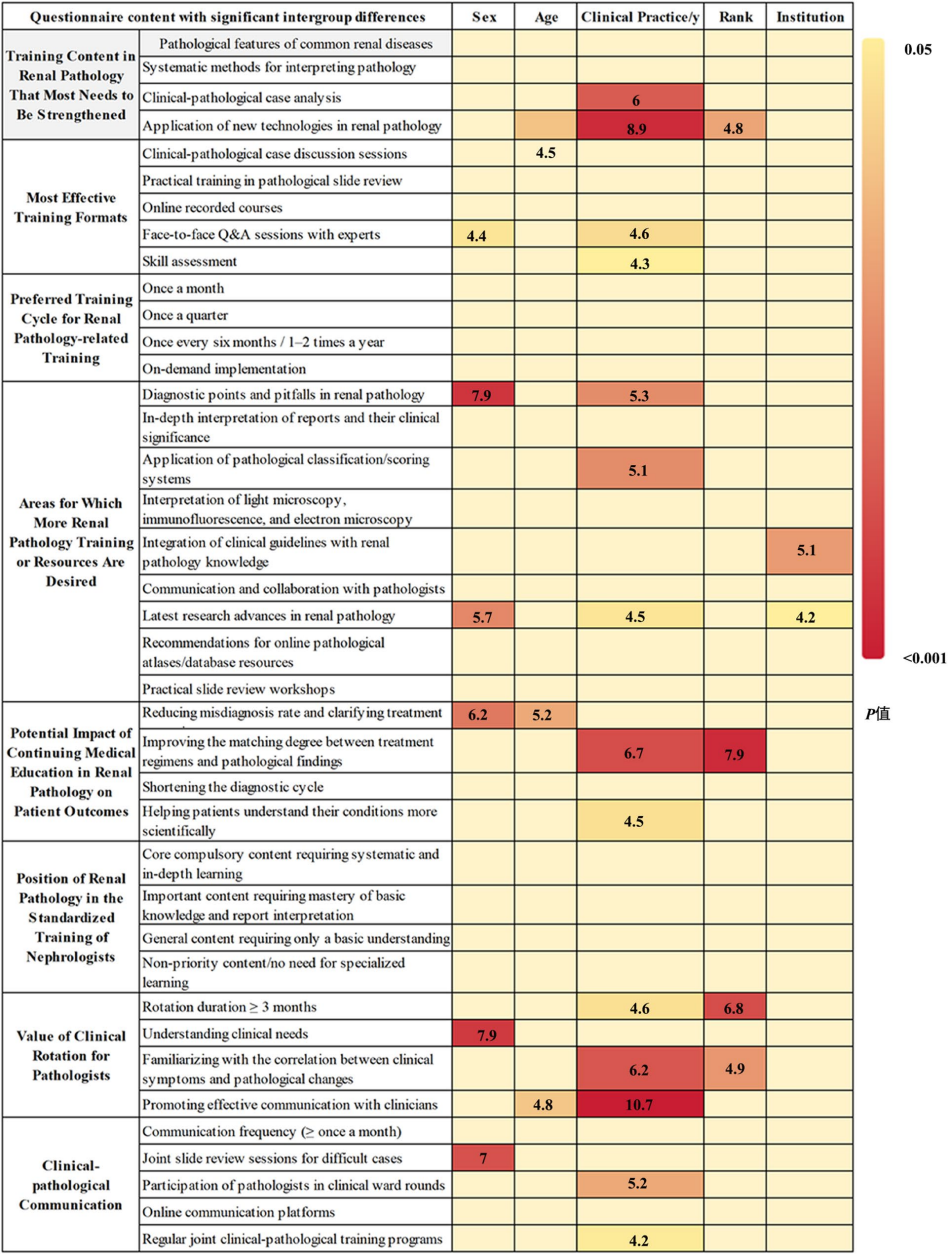
Importance, implementation methods and effective approaches of renal pathology in continuing medical education for nephrologists Note: On the one hand, this figure presents the main content of the questionnaire survey; on the other hand, it shows the results of the difference analysis regarding the varying perceptions or attitudes of nephrologists toward renal pathology-related survey content, stratified by gender, age, working years, professional title and affiliation. For gender, the comparison was made between male and female physicians; for age, between those aged ≤ 40 years and > 40 years; for working years, between physicians with ≤ 10 years and > 10 years of clinical practice; for professional title, between those with attending physician title or below and those with associate chief physician title or above; for affiliation, between physicians working in Grade A tertiary hospitals and those working in Grade B tertiary hospitals or below

A total of 224 physicians (87.5%) believed that training on the pathological features of common renal diseases should be strengthened. There are 215 physicians (84.0%) who emphasized the need for training on systematic methods for interpreting pathology reports, and 200 physicians (78.1%) considered clinical-pathological case analysis to be a key training content. And 160 physicians (62.5%) advocated for training on new renal pathology technologies such as immunofluorescence and electron microscopy. Particularly, compared with physicians with less than 10 years of clinical experience, those with over 10 years of clinical experience believed that training on new renal pathology technologies should be prioritized in future CME (*p* = 0.047, OR = 2.06, 95% CI = 1.01–4.20), and they also considered clinical-pathological case correlation analysis (*p* = 0.004, OR = 4.31, 95% CI = 1.57–11.81) to be an important training content (Fig. 4, Supplemental Tables 4 & 6).

Regarding training formats, clinical-pathological case discussion sessions were the most favored (222 physicians, 86.7%), followed by practical pathological slide review workshops (199 physicians, 77.7%). Female physicians (*p* = 0.019, OR = 1.88, 95% CI = 1.11–3.17) and those with over 10 years of clinical experience (*p* = 0.001, OR = 3.56, 95% CI = 1.64–7.71) showed a stronger preference for face-to-face expert Q&A sessions compared with their counterparts. As for the training cycle, 85 physicians (33.2%) supported monthly training, 85 physicians (33.2%) preferred quarterly training, 62 physicians (24.2%) opted for semi-annual training, and 24 physicians (9.4%) chose on-demand training upon the update of new technologies or guidelines. No significant differences were observed in the preferred training cycle among physicians with different genders, ages, clinical experience durations, professional titles, or affiliated institutions.

In terms of training content, 207 physicians (80.9%) expressed the need for training on the major diagnostic points and pitfalls of common and difficult renal diseases. Overall, 204 physicians (79.7%) desired in-depth training on the interpretation of renal biopsy reports and their clinical significance. And 175 physicians (68.4%) believed that training on the interpretation skills of light microscopy, immunofluorescence, and electron microscopy images was essential. In addition, some physicians chose other training content, including the detailed explanation and application of pathological classification/scoring systems, the integration of the latest clinical guidelines with renal pathology knowledge (61.7%), the latest advances in renal pathology research (47.3%), and recommendations for online pathological atlases/database resources (37.1%). Among these, female physicians (*p* = 0.006, OR = 2.14, 95% CI = 1.25–3.66) and those with over 10 years of clinical experience (*p* = 0.001, OR = 3.46, 95% CI = 1.68–7.13) were more inclined to desire training or resources related to the latest advances in renal pathology research. In addition, female physicians also expressed a stronger demand for training on the main diagnostic points of renal diseases and the interpretation of pathological grading systems (*p* = 0.003, OR = 2.79, 95% CI = 1.43–5.42) (Fig. 4, Supplemental Tables 4 & 6).

Regarding the role of renal pathology in the standardized training of nephrologists, 81 physicians (31.6%) considered it to be a core compulsory content requiring systematic and in-depth learning. And 139 physicians (54.3%) regarded it as an important content requiring mastery of basic knowledge and report interpretation skills. A total of 140 physicians (54.7%) believed that the optimal rotation duration in renal pathology should be more than 3 months, and this view was more widely supported by physicians holding titles at or above the level of Associate Chief Physician (*p* = 0.003, OR = 2.29, 95% CI = 1.08–4.83). The main value of clinical rotation for pathologists was recognized as understanding clinical needs (213 physicians, 83.2%), familiarizing with the correlation between clinical symptoms and pathological changes (211 physicians, 82.4%), and promoting communication between clinicians and pathologists (152 physicians, 59.4%). A total of 229 physicians (89.5%) supported a communication frequency of at least once a month between clinicians and pathologists. Joint slide review sessions for difficult cases were the most favored communication method (212 physicians, 82.8%), and this format was particularly popular among female physicians (*p* = 0.009, OR = 2.48, 95% CI = 1.25–4.90). In addition, 129 physicians (50.4%) supported regular joint clinical-pathological training programs, and this approach was more frequently chosen by physicians with over 10 years of clinical experience (*p* = 0.004, OR = 2.05, 95% CI = 1.03–4.08) (Fig. 4, Supplemental Tables 4 & 6).

## Discussion

### Continuing medical education in renal pathology is an inevitable trend

Renal pathological diagnosis serves as a critical cornerstone for clinicians to achieve accurate disease diagnosis, formulate treatment regimens, and evaluate patient prognosis [1, 2]. Therefore, proficient performance of pathological diagnosis for renal biopsy and solid mastery of renal pathology knowledge are essential to ensure reliable clinical diagnosis and treatment [13, 14]. Different from previous educational studies, this study focuses on the demands of nephrologists for CME in renal pathology. It elaborates on issues related to renal pathology CME from several aspects, including nephrologists’ attitudes toward renal pathology, the current status of CME and the problems required to be solved, as well as the value of third-party pathological testing and AI-assisted pathology at present. It should be noted that primary medical institutions still rely on third-party testing institutions. Regarding the clinical significance of AI-assisted pathology, the attitudes of clinicians, especially senior nephrologists, remain relatively negative. Of course, both in clinical practice and medical education, we need to recognize that AI should serve more as an auxiliary tool rather than a substitute for pathologists [15]. 

The analysis of this study revealed that female physicians exhibit a higher interest in learning renal pathology knowledge. And a greater proportion of physicians with more than 10 years of clinical experience believe that clinicians need to master the pathological features, pathological classification, and molecular pathology technologies of common diseases. Meanwhile, the main difficulties encountered by most nephrologists in interpreting renal pathology reports focus on clarifying the correlation between pathological descriptions and clinical symptoms or significance. Generally speaking, senior physicians have a deeper understanding of solving and improving clinical problems, encounter more complex cases, and take on more responsibilities for discipline development; And they also reported the difficulties in communicating with pathologists in clinical work, the main reason for this phenomenon is more likely to be the shortage of pathologists and pathological resources. To a certain extent, the above results also remind us of the importance of clinical pathology interpretation in the CME of nephrologists [16, 17]. 

The main channels for nephrologists to acquire and update renal pathology knowledge include attending academic conferences, thematic lectures, and specialized pathological training programs. However, the current CME in renal pathology is confronted with several urgent problems to be solved, such as the mismatch between training content and individual physicians’ needs, the singular training format lacking practical operation sessions, the absence of online courses for physicians who cannot attend offline training due to time constraints, and insufficient funding support. Therefore, it is important to carry out CME of renal pathology for nephrologists, and its main forms could include case-based clinical-pathological discussions, academic conferences, thematic lectures, and other related pathological training activities. In general, in renal pathology teaching, priority should be given to ensuring that physicians have a solid grasp of basic theoretical knowledge of renal pathology, while diverse approaches should be adopted to stimulate the learning initiative of further education physicians. In addition, the development and improvement of clinical-pathological correlation analysis and discussions are crucial, as they are of great significance in helping further education physicians deepen their understanding of renal diseases, and strengthen their abilities in comprehensive analysis and problem-solving [18]. 

### Targeted pathological training oriented to clinical pain points and job requirements is the core format

Regarding training formats and demands, in addition to clinical-pathological case discussions and practical pathological slide review sessions, female physicians demonstrate a stronger interest in face-to-face expert Q&A sessions. Correspondingly, female physicians also express a greater desire to update their knowledge on the main diagnostic points and pitfalls of common and challenging renal diseases, as well as the latest advances in renal pathology research [15]. Furthermore, they prefer clinical-pathological communication in the form of joint slide review sessions for difficult cases. Previous educational studies have also indicated that the case-based teaching method exhibits distinct advantages in renal pathology education, as it can effectively improve the quality of clinical teaching and is worthy of promotion and application in clinical education [19, 20]. 

In terms of age stratification, physicians under 40 years old primarily update their renal pathology knowledge through medical school courses and resident/specialist standardized training, which may be attributed to the fact that a portion of these physicians are currently undergoing standardized training. This also reminds us that renal pathology training can be incorporated into the resident/specialist training program for nephrologists. In contrast, physicians over 40 years old are more eager to receive enhanced training on the application of new renal pathology technologies, such as immunofluorescence and electron microscopy [21]. Physicians with different lengths of clinical experience also exhibit varying tendencies toward the value of renal pathology and the preferred methods and content of CME. First, physicians with over 10 years of clinical experience attach greater importance to the role of renal pathology in clinical diagnosis and treatment, the necessity for nephrologists to master pathological knowledge, and the ability to independently interpret pathology reports, which further reflects the close correlation between renal pathology and clinical practice [14]. And these physicians are more likely to learn and update their pathological knowledge by attending academic conferences/lectures/training courses, reading professional books and journal articles, and participating in case discussions.

Moreover, this group of physicians identifies the main problems in current CME as a singular training format lacking online options and a lack of targeted training content. They also express a strong desire to update their knowledge on the pathological diagnosis of common and difficult renal diseases, the detailed explanation and application of pathological classification/scoring systems, and the latest advances in renal pathology research, such as digital renal pathology [15]. In addition, clinical ward rounds with the participation of pathologists and regular joint clinical-pathological training programs should be established as the main forms of clinical-pathological communication. For physicians with different professional titles, those at or above the level of Associate Chief Physician are more eager to strengthen effective communication and joint slide review with pathologists, as well as obtain targeted training and knowledge updates on new pathological technologies. Studies have shown that the digital slide-based case teaching method yields significantly better results in nephrology clinical education, compared with the traditional pathological ward round model involving digital slides reading, as it can not only enrich teaching content but also improve teaching quality [11, 22]. 

Due to objective constraints such as a shortage of technical personnel and a lack of specimen processing equipment [23], the implementation of renal pathology services in primary medical institutions is severely limited. Therefore, their core demands are access to rapid channels for sending specimens to external institutions and telepathology consultation support. An experimental study analyzed and compared three pathological slide reading methods, concluding that telepathology slide reading achieves an accuracy rate that basically meets diagnostic and teaching requirements and is highly feasible in practice [24]. Based on the needs of nephrologists in primary care settings, the value of telepathology slide reading can be fully leveraged. On the one hand, the time allocated to weekly clinical-pathological slide review sessions can be fully utilized to enhance the effectiveness of pathology learning. On the other hand, systematic renal pathology lectures can be organized to systematize the knowledge of renal pathology.

In summary, nephrologists with different genders, ages, lengths of clinical experience, professional titles, and affiliated institutions exhibit significant differences in their demand for renal pathology knowledge, as well as their preferred training methods and content. Therefore, future renal pathology training programs for nephrologists should be designed in a targeted manner tailored to the specific needs of different physician groups. Of course, the occurrence of this difference would not be excluded from social desirability bias, and further research is expected to confirm whether these differences truly reflect the actual educational needs.

### Conclusion

Through a survey of nephrologists, this study found that nearly all physicians recognize the absolute value of renal pathology in disease diagnosis, treatment, and prognosis evaluation [25], and they also express a strong desire to supplement and update their renal pathology knowledge through CME. First, it is imperative to formulate training programs based on the needs of different categories of nephrologists, including case-based clinical-pathological discussions, practical pathological slide review sessions, and training programs focusing on the latest advances in renal pathology research and new pathological technologies. Second, physicians with extensive clinical experience tend to prioritize knowledge updates on the close correlation between renal pathology and clinical practice, as well as clinical-pathological communication. Finally, the value of telepathology slide reading should be fully exploited in the knowledge update system for nephrologists in primary care settings.

Of course, this study has certain limitations. Firstly, the questionnaire design has drawbacks such as excessive content and insufficient specificity, which may have prevented a full elucidation of the problems existing in CME in renal pathology and the precise needs of nephrologists at all levels. Secondly, regarding the questionnaire content, it lacks formal instrument validation apart from internal consistency analysis and expert review. Thirdly, we adopted convenience sampling via WeChat, which may introduce selection bias and result in the absence of a reliable response rate. For instance, respondents who have a stronger interest in renal pathology and related education, as well as those from highly informatized hospitals, would be over-represented. Fourthly, although the survey included physicians from medical institutions at all levels, the sample size is relatively limited, and the generalizability of the findings remains to be verified. Fifthly, although we adjusted for the confounding effects of gender, age, length of service, professional title, and hospital level using multivariate logistic regression analysis, we did not account for the influence of physicians’ different nephrology subspecialties, which could be further addressed in future educational research. Sixthly, to facilitate analysis and result presentation, certain Likert scale items were treated as dichotomous variables for statistical analysis. Seventhly, this study conducted multiple subgroup analyses, which may increase the risk of Type I error inflation. Therefore, conclusions drawn from these subgroup comparisons should be interpreted comprehensively and in combination with clinical relevance. Finally, certain levels of social desirability bias and systematic errors resulting from self-reported data were unavoidable in this study. In conclusion, the conclusions of such studies should be interpreted dialectically and combined with actual clinical scenarios, avoiding overinterpretation of statistical results. Future studies are desired to be more targeted, with more in-depth questionnaire content and a larger sample of nephrologists, thereby providing more comprehensive and detailed references for CME in renal pathology and clinical-pathological correlation for nephrologists.

## Supplementary Information

Supplementary Material 1.

Supplementary Material 2.

Supplementary Material 3.

Supplementary Material 4.

Supplementary Material 5.

Supplementary Material 6.

Supplementary Material 7.

Supplementary Material 8.

## Data Availability

The data investigated and analyzed during this study are available from the corresponding author upon reasonable request.
