# Supplementary material for: Continuing medical education in renal pathology: current practices and needs among nephrologists
Source: BMC Med Educ. 2026 Feb 12;26:441. doi: 10.1186/s12909-026-08798-4 (PMC12997942; doi:10.1186/s12909-026-08798-4)
Supplement: Supplementary file 1 — Supplementary Material 1. [file 12909_2026_8798_MOESM1_ESM.docx]

**Survey Questionnaire**

| Number | Title | Option |
| --- | --- | --- |
| 1 | Gender | □ Male □ Female |
| 2 | Age | □ < 30 years old □ 30–40 years old □ 41–50 years old □ > 50 years old |
| 3 | Years of Clinical Practice | □ < 5 years □ 5–10 years □ 11–20 years □ > 20 years |
| 4 | Professional Title | □ Resident Physician □ Attending Physician □ Associate Chief Physician □ Chief Physician |
| 5 | Current Primary Affiliation | □ Grade III Class A Hospital □ Grade III Class B/Other Grade III Hospitals □ Grade II Hospital □ Primary Medical Institution (Community Health Service Center/Township Health Center) □ Other (Please specify): _________ |
| 6 | Department/Subspecialty | □ Nephrology Ward □ Nephrology Outpatient Clinic □ Hemodialysis/Peritoneal Dialysis Center □ Kidney Transplantation Center □ Renal Pathology □ Other ______ |
| 7 | Average Weekly Volume of Renal Disease Patients Consulted or Managed | □ < 10 cases □ 10–30 cases □ 31–50 cases □ > 50 cases |
| 8 | Annual Frequency of Participating in Renal Pathology Report Interpretation | □ Almost never □ < 10 reports/year □ 10–50 reports/year □ 51–100 reports/year □ > 100 reports/year |
| 9 | Importance of Mastering Basic Renal Pathology Knowledge (e.g., common pathological types, light microscopy/electron microscopy/immunofluorescence findings) for Nephrologists | □ Extremely important □ Quite important □ Moderately important □ Slightly important □ Not important at all |
| 10 | Importance of the Ability to Independently Interpret Core Information of Renal Biopsy Reports (e.g., pathological diagnosis, grading and scoring systems) for Nephrologists | □ Extremely important □ Quite important □ Moderately important □ Slightly important □ Not important at all |
| 11 | Desired Depth of Renal Pathology Knowledge for Clinicians (Non-Pathologists) (Single Choice) | □ Basic pathological terminology (e.g., "mesangial proliferation", "crescent formation") □ Pathological features of common diseases (e.g., IgA nephropathy, diabetic nephropathy) □ Differential diagnosis of complex pathological types (e.g., lupus nephritis classification, membranous nephropathy staging) □ Clinical significance of immunofluorescence/electron microscopy results |
| 12 | Main Difficulties in Interpreting Renal Pathology Reports (Multiple Choices Allowed) | □ Opaque pathological terminology □ Difficulty in correlating pathological descriptions with clinical manifestations □ Unclear clinical significance of different pathological changes □ Lack of effective communication with pathologists □ Other ______ |
| 13 | Core Needs of Primary Medical Institutions for Renal Pathology (Multiple Choices Allowed) | □ Channels for rapid specimen transportation to external institutions □ Simplified pathology reports □ Telepathology consultation support □ Renal pathology knowledge training tailored for primary institutions □ Other ______ |
| 14 | Main Restrictive Factors for Primary Institutions to Carry Out Renal Pathology-Related Work (Multiple Choices Allowed) | □ Lack of specimen processing equipment □ Insufficient technical personnel □ High cost of external specimen transportation □ Unsmooth cooperation mechanism with pathology departments of superior hospitals □ Other ______ |
| 15 | Does Your Institution Rely on Third-Party Pathological Testing Institutions? | □ Yes (regularly send specimens to superior hospitals, e.g., primary hospitals to tertiary hospitals) □ No (equipped with its own pathology department) □ Only send difficult or specific cases for external testing |
| 16 | Main Factors Influencing Trust in Third-Party Institutions (Multiple Choices Allowed) | □ Testing qualifications (e.g., certified laboratories) □ Timeliness of reports □ Ability to provide clinical interpretation suggestions □ Reasonableness of prices |
| 17 | Potential Value of AI in Renal Pathology (Multiple Choices Allowed) | □ Rapid preliminary screening (e.g., identifying typical lesions, reducing manual workload) □ Quantitative analysis (e.g., automatic counting of glomerulosclerosis ratio) □ Assisting primary physicians in report interpretation (e.g., AI generates simplified conclusions) □ No significant value (reliance on pathologists' experience is more reliable) |
| 18 | Main Concerns Regarding AI Application in Renal Pathology (Multiple Choices Allowed) | □ Misdiagnosis risk (especially for rare/complex cases) □ Data privacy leakage (patients' pathological images) □ Over-reliance on AI, weakening physicians' subjective judgment ability □ Lack of unified standards, leading to inconsistent results among different AI systems |
| 19 | Main Channels for Learning and Updating Renal Pathology Knowledge (Multiple Choices Allowed) | □ Medical school courses □ Resident/specialist standardized training □ Attending academic conferences/lectures/training courses □ Reading professional books and journal articles □ Online learning resources (e.g., online courses, databases, pathological atlases) □ Communication with pathologists/joint slide review □ Case conferences (intra-departmental/MDT) □ Self-study □ Other (Please specify): _________ |
| 20 | Current Frequency of Participating in Renal Pathology-Related Training (e.g., academic conferences, slide review sessions) | □ ≥ 3 times/year □ 1–2 times/year □ Rarely participate □ Never participate |
| 21 | Improvement of Capabilities After Participating in Renal Pathology-Related Training (Multiple Choices Allowed) | □ Accuracy of pathology report interpretation □ Ability of clinical-pathological correlation analysis □ Confidence in diagnosing and treating complex cases □ Efficiency of communication with pathologists □ No obvious improvement □ Other ______ |
| 22 | Most Needed Content to Be Strengthened in Renal Pathology Training (Multiple Choices Allowed) | □ Pathological features of common renal diseases □ Systematic methods for interpreting pathology reports □ Clinical-pathological case analysis □ Application of new renal pathology technologies (e.g., immunofluorescence, electron microscopy) □ Other ______ |
| 23 | Main Difficulties in Participating in Renal Pathology Continuing Medical Education (Multiple Choices Allowed) | □ Time conflicts (busy clinical work, difficulty in being absent) □ Training resources concentrated in large cities, inconvenient for primary institution staff to participate □ Mismatch between training content and personal needs (e.g., content is too basic for tertiary hospital physicians, too complex for primary staff) □ Lack of funding support (e.g., training fees, travel expenses) □ Single training format (e.g., only online, lacking practical operation) □ Other ______ |
| 24 | Main Problems Existing in Current Renal Pathology Continuing Medical Education (Multiple Choices Allowed) | □ Low training frequency (e.g., only 1–2 times a year) □ Disconnection between content and clinical practice (e.g., pure theory, lack of case analysis) □ Single training format (e.g., only offline lectures, no online playback) □ Lack of targeting (e.g., failing to distinguish the needs of physicians with different professional titles) □ Absence of assessment mechanisms (no feedback on learning outcomes after training) □ Other ______ |
| 25 | Most Effective Training Formats (Multiple Choices Allowed) | □ Clinical-pathological case discussion sessions □ Practical renal pathology slide review workshops □ Online recorded courses □ Face-to-face expert Q&A sessions □ Skill assessment □ Other ______ |
| 26 | Desired Training Cycle for Renal Pathology-Related Programs | □ Once a quarter □ Once every six months □ 1–2 times a year □ On-demand (e.g., upon the update of new technologies/guidelines) |
| 27 | Areas in Need of More Renal Pathology Training or Resources (Multiple Choices Allowed) | □ Key diagnostic points and pitfalls of common and difficult renal diseases □ In-depth interpretation of renal biopsy reports and their clinical significance □ Detailed explanation and application of pathological classification/scoring systems □ Interpretation skills of light microscopy, immunofluorescence and electron microscopy images □ Integration of the latest clinical guidelines with renal pathology knowledge (e.g., KDIGO guideline interpretation) □ Strategies for better communication and collaboration with pathologists □ Latest advances in renal pathology research □ Recommendations for online pathological atlases/database resources □ Practical slide review workshops □ Other (Please specify): _________ |
| 28 | Potential Impact of Systematic Renal Pathology Continuing Medical Education on Patient Outcomes (Multiple Choices Allowed) | □ Reduce misdiagnosis rate and unnecessary treatment □ Improve the matching degree between treatment plans and pathological types, enhancing curative effect □ Shorten diagnostic cycle and reduce patient waiting time □ Help patients understand their conditions more scientifically □ No obvious impact |
| 29 | Position of Renal Pathology in the Standardized Training (Residency/Specialty Training) for Nephrologists | □ Core compulsory content, requiring systematic and in-depth learning □ Important content, requiring mastery of basic knowledge and report interpretation skills □ Moderate content, requiring basic understanding only □ Non-priority content □ No need for dedicated learning |
| 30 | Optimal Duration of Renal Pathology Rotation | □ 1–3 months □ 3–6 months □ 6–12 months □ On-demand rotation |
| 31 | Main Value of Clinical Rotation for Pathologists (Multiple Choices Allowed) | □ Understand clinical needs, making reports more suitable for diagnosis and treatment decisions □ Familiarize with the correlation between clinical manifestations and pathological changes, reducing misdiagnosis □ Promote communication rapport with clinicians □ No obvious value |
| 32 | Frequency of Communication Between Clinicians and Pathologists | □ ≥ Once a week (e.g., joint case discussions) □ 1–2 times a month □ Only communicate when encountering difficult cases □ Almost no communication |
| 33 | Most Effective Clinical-Pathological Communication Methods (Multiple Choices Allowed) | □ Joint slide review sessions for difficult cases □ Pathologists participating in clinical ward rounds □ Online communication platforms (e.g., clinical suggestions attached to pathology reports) □ Regular joint clinical-pathological training programs □ Other ______ |
| 34 | Role of Renal Biopsy Pathology Results in Clinical Practice | 1. Clarifying disease diagnosis: □ Extremely important □ Quite important □ Moderately important □ Slightly important □ Not important at all |
|  |  | 2. Assessing disease activity and severity: □ Extremely important □ Quite important □ Moderately important □ Slightly important □ Not important at all |
|  |  | 3. Predicting disease progression and prognosis: □ Extremely important □ Quite important □ Moderately important □ Slightly important □ Not important at all |
|  |  | 4. Guiding individualized treatment plan selection: □ Extremely important □ Quite important □ Moderately important □ Slightly important □ Not important at all |
|  |  | 5. Evaluating treatment response: □ Extremely important □ Quite important □ Moderately important □ Slightly important □ Not important at all |
|  |  | 6. Determining eligibility for clinical trials: □ Extremely important □ Quite important □ Moderately important □ Slightly important □ Not important at all |
| 35 | Importance of Renal Pathology for Specific Renal Diseases | 1. Primary glomerular diseases (e.g., IgA nephropathy): □ Extremely important □ Quite important □ Moderately important □ Slightly important □ Not important at all |
|  |  | 2. Secondary glomerular diseases (e.g., diabetic nephropathy): □ Extremely important □ Quite important □ Moderately important □ Slightly important □ Not important at all |
|  |  | 3. Acute kidney injury (when etiology needs to be differentiated): □ Extremely important □ Quite important □ Moderately important □ Slightly important □ Not important at all |
|  |  | 4. Chronic kidney disease (when assessing progression risk): □ Extremely important □ Quite important □ Moderately important □ Slightly important □ Not important at all |
|  |  | 5. Post-kidney transplantation complications (e.g., rejection): □ Extremely important □ Quite important □ Moderately important □ Slightly important □ Not important at all |
| 36 | Open-Ended Question | What do you think is the most core value of "renal pathology continuing medical education" for nephrologists? What is the most urgent specific problem that needs to be solved? |
