# Supplementary material for: Continuing medical education in renal pathology: current practices and needs among nephrologists
Source: BMC Med Educ. 2026 Feb 12;26:441. doi: 10.1186/s12909-026-08798-4 (PMC12997942; doi:10.1186/s12909-026-08798-4)
Supplement: Supplementary file 2 — Supplementary Material 2. [file 12909_2026_8798_MOESM2_ESM.docx]

**Supplemental Table 1. Analysis of Questionnaire Results on the Importance and Existing Problems of Renal Pathology**

| **Observation indicators** | **All (n=256)** | **Sex** | | | **Age** | | | **Working time** | | | **Medical Professional Title** | | | Affiliation | | | **Report Interpretation Volume** | | |
| --- | --- | --- | --- | --- | --- | --- | --- | --- | --- | --- | --- | --- | --- | --- | --- | --- | --- | --- | --- |
|  |  | **Male（n=111）** | **Female (n=145)** | **p** | **≤40y（n=180）** | **＞40y（n=76）** | **p** | **≤10y（n=151）** | **＞10y（n=105）** | **p** | **Attending Physician and Below（n=188）** | **Associate Chief Physician and Above（n=68）** | **p** | **Grade A Tertiary Hospital（n=148）** | **Grade B Tertiary Hospital and Below（n=108）** | **p** | **≤10 cases/year（n=180）** | **＞10 cases/year（n=76）** | **p** |
| **Analysis of Importance and Existing Problems** | | | | | | | | | | | | | | | | | | | |
| Importance of Mastering Basic Knowledge of Renal Pathology (Extremely Important) | 193 (75.4) | **80** (72.1) | **113** (77.9) | **ns** | **132** (73.3) | **61** (80.3) | **ns** | **106** (70.2) | **87** (82.9) | **0.027** | **139** (73.9) | **54** (79.4) | **ns** | 117 (79.1) | 76 (70.4) | **ns** | 134 (74.4) | 59 (77.6) | **ns** |
| Ability to Independently Interpret Pathology Reports (Extremely Important) | 180 (70.3) | **67** (60.4) | **113** (77.9) | **0.004** | **123** (68.3) | **57** (75.0) | **ns** | **98** (64.9) | **82** (78.1) | **0.026** | **131** (69.7) | **49** (72.1) | **ns** | 104 (70.3) | 76 (70.4) | **ns** | 123 (68.3) | 57 (75.0) | **ns** |
| **Analysis of Importance and Existing Problems** | | | | | | | | | | | | | | | | | | | |
| Clarifying Disease Diagnosis (Extremely Important) | 199 (77.7) | **76** (68.5) | **123** (84.8) | **0.002** | **136** (75.6) | **63** (82.9) | **ns** | **109** (72.2) | **90** (85.7) | **0.014** | **144** (76.6) | **55** (80.9) | **ns** | 121 (81.8) | 78 (72.2) | **ns** | 140 (77.8) | 59 (77.6) | **ns** |
| Assessing Disease Activity and Severity (Extremely Important) | 190 (74.2) | **75** (67.6) | **115** (79.3) | **0.043** | **130** (72.2) | **60** (78.9) | **ns** | **106** (70.2) | **84** (80.0) | **0.083** | **135** (71.8) | **55** (80.9) | **ns** | 117 (79.1) | 73 (67.6) | **0.043** | 129 (71.7) | 61 (80.3) | **ns** |
| Predicting Disease Progression and Prognosis (Extremely Important) | 185 (72.3) | **70** (63.1) | **115** (79.3) | **0.005** | **125** (69.4) | **60** (78.9) | **ns** | **101** (66.9) | **84** (80.0) | **0.023** | **130** (69.1) | **55** (80.9) | **0.082** | 116 (78.4) | 69 (63.9) | **0.011** | 125 (69.4) | 60 (78.9) | **ns** |
| Guiding Individualized Treatment Regimen Selection (Extremely Important) | 187 (73.0) | **71** (64.0) | **116** (80.0) | **0.005** | **128** (71.1) | **59** (77.6) | **ns** | **103** (68.2) | **84** (80.0) | **0.045** | **134** (71.3) | **53** (77.9) | **ns** | 116 (78.4) | 71 (65.7) | **0.032** | 129 (71.7) | 58 (76.3) | **ns** |
| Evaluating Treatment Response (Extremely Important) | 180 (70.3) | **67** (60.4) | **113** (77.9) | **0.004** | **121** (67.2) | **59** (77.6) | **0.102** | **98** (64.9) | **82** (78.1) | **0.026** | **129** (68.6) | **51** (75.0) | **ns** | 109 (73.6) | 71 (65.7) | **ns** | 123 (68.3) | 57 (75.0) | **ns** |
| Determining Eligibility for Clinical Trials (Extremely Important) | 160 (62.5) | **62** (55.9) | **98** (67.6) | **ns** | **112** (62.2) | **48** (63.2) | **ns** | **89** (58.9) | **71** (67.6) | **0.190** | **116** (61.7) | **44** (64.7) | **ns** | 95 (64.2) | 65 (60.2) | **ns** | 110 (61.1) | 50 (65.8) | **ns** |
| **Importance of Renal Pathology in Various Diseases** | | | | | | | | | | | | | | | | | | | |
| Primary Glomerular Diseases (e.g., IgA Nephropathy) (Extremely Important) | 183 (71.5) | **76** (68.5) | **107** (73.8) | **ns** | **123** (68.3) | **60** (78.9) | **ns** | **98** (64.9) | **85** (81.0) | **0.005** | **128** (68.1) | **55** (80.9) | **0.059** | 107 (72.3) | 76 (70.4) | **ns** | 124 (68.9) | 59 (77.6) | **ns** |
| Secondary Glomerular Diseases (e.g., Diabetic Nephropathy) (Extremely Important) | 156 (60.9) | **63** (56.8) | **93** (64.1) | **ns** | **101** (56.1) | **55** (72.4) | **0.017** | **83** (55.0) | **73** (69.5) | **0.02** | **110** (58.5) | **46** (67.6) | **ns** | 90 (60.8) | 66 (61.1) | **ns** | 104 (57.8) | 52 (68.4) | **ns** |
| Acute Kidney Injury (AKI) (When Etiological Differentiation is Required) (Extremely Important) | 160 (62.5) | **64** (57.7) | **96** (66.2) | **ns** | **108** (60.0) | **52** (68.4) | **ns** | **88** (58.3) | **72** (68.6) | **0.115** | **113** (60.1) | **47** (69.1) | **ns** | 92 (62.2) | 68 (63.0) | **ns** | 109 (60.6) | 51 (67.1) | **ns** |
| Chronic Kidney Disease (CKD) (When Assessing Progression Risk) (Extremely Important) | 148 (57.8) | **61** (55.0) | **87** (60.0) | **ns** | **97** (53.9) | **51** (67.1) | **0.054** | **82** (54.3) | **66** (62.9) | **ns** | **109** (58.0) | **39** (57.4) | **ns** | 83 (56.1) | 65 (60.2) | **ns** | 106 (58.9) | 42 (55.3) | **ns** |
| Post-Kidney Transplant Complications (e.g., Rejection) (Extremely Important) | 168 (65.6) | **64** (57.7) | **104** (71.7) | **ns** | **115** (63.9) | **53** (69.7) | **ns** | **96** (63.6) | **72** (68.6) | **ns** | **121** (64.4) | **47** (69.1) | **ns** | 100 (67.6) | 68 (63.0) | **ns** | 118 (65.6) | 50 (65.8) | **ns** |
| **Depth of Renal Pathology Knowledge Required for Non-Pathologist Clinicians** | | | | | | | | | | | | | | | | | | | |
| Basic Pathological Terminology (e.g., "Mesangial Proliferation", "Crescent Formation") | 208 (81.3) | **83** (74.8) | **125** (86.2) | **0.024** | **144** (80.0) | **64** (84.2) | **ns** | **119** (78.8) | **89** (84.8) | **ns** | **150** (79.8) | **58** (85.3) | **ns** | 123 (83.1) | 85 (78.7) | **ns** | 147 (81.7) | 61 (80.3) | **ns** |
| Pathological Features of Common Diseases (e.g., IgA Nephropathy, Diabetic Nephropathy) | 231 (90.2) | **97** (87.4) | **134** (92.4) | **ns** | **162** (90.0) | **69** (90.8) | **ns** | **129** (85.4) | **102** (97.1) | **0.002** | **166** (88.3) | **65** (95.6) | **ns** | 133 (89.9) | 98 (90.7) | **ns** | 163 (90.6) | 68 (89.5) | **ns** |
| Differentiation of Complex Pathological Types (e.g., Lupus Nephritis Classification, Membranous Nephropathy Staging) | 173 (67.6) | **74** (66.7) | **99** (68.3) | **ns** | **117** (65.0) | **56** (73.7) | **ns** | **89** (58.9) | **94** (89.5) | **<0.001** | **121** (64.4) | **52** (76.5) | **0.072** | 101 (68.2) | 72 (66.7) | **ns** | 118 (65.6) | 55 (72.4) | **ns** |
| Clinical Significance of Immunofluorescence/Electron Microscopy Results | 161 (62.9) | **56** (50.5) | **105** (72.4) | ＜0.001 | **108** (60.0) | **53** (69.7) | **ns** | **81** (53.6) | **80** (76.2) | **<0.001** | **112** (59.6) | **49** (72.1) | **0.079** | 102 (68.9) | 59 (54.6) | **ns** | 112 (62.2) | 49 (64.5) | **ns** |
| **Main Difficulties in Interpreting Renal Pathology Reports** | | | | | | | | | | | | | | | | | | | |
| Opaque and Hard-to-Understand Pathological Terminology | 139 (54.3) | **59** (53.2) | **80** (55.2) | **ns** | **95** (52.8) | **44** (57.9) | **ns** | **82** (54.3) | **57** (54.3) | **ns** | **103** (54.8) | **36** (52.9) | **ns** | 73 (49.3) | 66 (61.1) | **0.075** | 105 (58.3) | 34 (44.7) | **0.055** |
| Difficulty in Correlating Pathological Descriptions with Clinical Symptoms | 182 (71.1) | **77** (69.4) | **105** (72.4) | **ns** | **133** (73.9) | **49** (64.5) | **ns** | **109** (72.2) | **73** (69.5) | **ns** | **136** (72.3) | **46** (67.6) | **ns** | 97 (65.5) | 85 (78.7) | **0.026** | 134 (74.4) | 48 (63.2) | **ns** |
| Unclear Clinical Significance of Different Pathological Changes | 182 (71.1) | **74** (66.7) | **108** (74.5) | **ns** | **131** (72.8) | **51** (67.1) | **ns** | **105** (69.5) | **77** (73.3) | **ns** | **130** (69.1) | **52** (76.5) | **ns** | 110 (74.3) | 72 (66.7) | **ns** | 128 (71.1) | 54 (71.1) | **ns** |
| Lack of Effective Communication with Pathologists | 139 (54.3) | **57** (51.4) | **82** (56.6) | **ns** | **93** (51.7) | **46** (60.5) | **ns** | **70** (46.4) | **69** (65.7) | **0.002** | **94** (50.0) | **45** (66.2) | **0.024** | 80 (54.1) | 59 (54.6) | **ns** | 97 (53.9) | 42 (55.3) | **ns** |
| **ns, not significant;** | | | | | | | | | | | | | | | | | | | |
