# Supplementary material for: Continuing medical education in renal pathology: current practices and needs among nephrologists
Source: BMC Med Educ. 2026 Feb 12;26:441. doi: 10.1186/s12909-026-08798-4 (PMC12997942; doi:10.1186/s12909-026-08798-4)
Supplement: Supplementary file 3 — Supplementary Material 3. [file 12909_2026_8798_MOESM3_ESM.docx]

**Supplemental Table 2. Analysis of Questionnaire Results on the Current Status, Problems and Difficulties of Renal Pathology-related Training**

|  | **All (n=256)** | **Sex** | | | **Age** | | | **Working time** | | | **Medical Professional Title** | | | **Affiliation** | | | **Report Interpretation Volume** | | |
| --- | --- | --- | --- | --- | --- | --- | --- | --- | --- | --- | --- | --- | --- | --- | --- | --- | --- | --- | --- |
|  |  | **Male（n=111）** | **Fema;e (n=145)** | **p** | **≤40y（n=180）** | **＞40y（n=76）** | **p** | **≤10 years（n=151）** | **＞10years（n=105）** | **p** | **Attending Physician and Below（n=188）** | **Associate Chief Physician and Above（n=68）** | **p** | **Grade A Tertiary Hospital（n=148）** | **Grade B Tertiary Hospital and Below（n=108）** | **p** | **≤10 cases/year（n=180）** | **＞10 cases/year （n=76）** | **p** |
| Current Status, Problems and Difficulties of Renal Pathology-related Training | | | | | | | | | | | | | | | | | | | |
| Through which channels do you mainly learn and update renal pathology knowledge? | | | | | | | | | | | | | | | | | | | |
| Medical School Courses | 149 (58.2) | 61 (55.0) | 88 (60.7) | ns | 113 (62.8) | 36 (47.4) | 0.027 | 93 (61.6) | 56 (53.3) | ns | 117 (62.2) | 32 (47.1) | 0.032 | 5 (57.4) | 64 (59.3) | ns | 106 (58.9) | 43 (56.6) | ns |
| Resident/Specialist Standardized Training | 145 (56.6) | 59 (53.2) | 86 (59.3) | ns | 117 (65.0) | 28 (36.8) | ＜0.001 | 99 (65.6) | 46 (43.8) | 0.001 | 120 (63.8) | 25 (36.8) | <0.001 | 76 (51.4) | 69 (63.9) | 0.055 | 100 (55.6) | 45 (59.2) | ns |
| Attending Academic Conferences/Lectures/Training Programs | 196 (76.6) | 87 (78.4) | 109 (75.2) | ns | 134 (74.4) | 62 (81.6) | ns | 103 (68.2) | 93 (88.6) | <0.001 | 137 (72.9) | 59 (86.8) | ns | 110 (74.3) | 86 (79.6) | ns | 134 (74.4) | 62 (81.6) | ns |
| Reading Professional Books and Journal Literatures | 159 (62.1) | 65 (58.6) | 94 (64.8) | ns | 110 (61.1) | 49 (64.5) | ns | 81 (53.6) | 78 (74.3) | 0.001 | 111 (59.0) | 48 (70.6) | 0.109 | 90 (60.8) | 69 (63.9) | ns | 102 (56.7) | 57 (75.0) | 0.007 |
| Online Learning Resources (e.g., Online Courses, Databases, Pathology Atlases) | 156 (60.9) | 71 (64.0) | 85 (58.6) | ns | 112 (62.2) | 44 (57.9) | ns | 84 (55.6) | 72 (68.6) | 0.038 | 109 (58.0) | 47 (69.1) | 0.113 | 86 (58.1) | 70 (64.8) | ns | 102 (56.7) | 54 (71.1) | 0.036 |
| Communication/Joint Slide Review with Pathologists | 104 (40.6) | 43 (38.7) | 61 (42.1) | ns | 66 (36.7) | 38 (50.0) | 0.052 | 52 (34.4) | 52 (49.5) | 0.02 | 69 (36.7) | 35 (51.5) | 0.043 | 60 (40.5) | 44 (40.7) | ns | 64 (35.6) | 40 (52.6) | 0.012 |
| Case Discussions (Departmental/MDT) | 106 (41.4) | 45 (40.5) | 61 (42.1) | ns | 69 (38.3) | 37 (48.7) | 0.129 | 53 (35.1) | 53 (50.5) | 0.015 | 71 (37.8) | 35 (51.5) | 0.062 | 62 (41.9) | 44 (40.7) | ns | 69 (38.3) | 37 (48.7) | ns |
| Self-study | 75 (29.3) | 37 (33.3) | 38 (26.2) | ns | 52 (28.9) | 23 (30.3) | ns | 39 (25.8) | 36 (34.3) | ns | 55 (29.3) | 20 (29.4) | ns | 41 (27.7) | 34 (31.5) | ns | 52 (28.9) | 23 (30.3) | ns |
| Do you currently participate in renal pathology-related training (e.g., academic conferences, pathology slide seminars)? | | | | | | | | | | | | | | | | | | | |
| ≥ 1 Time/Year | 136 (53.1) | 65 (58.6) | 71 (49.0) | 0.132 | 99 (55.0) | 37 (48.7) | ns | 91 (60.3) | 45 (42.9) | 0.007 | 105 (55.9) | 31 (45.6) | ns | 84 (56.8) | 52 (48.1) | ns | 82 (45.6) | 54 (71.1) | <0.001 |
| Do you think the following abilities have improved after attending renal pathology-related training? | | | | | | | | | | | | | | | | | | | |
| Accuracy of Pathology Report Interpretation | 199 (77.7) | 82 (73.9) | 117 (80.7) | ns | 138 (76.7) | 61 (80.3) | ns | 112 (74.2) | 87 (82.9) | ns | 144 (76.6) | 55 (80.9) | ns | 119 (80.4) | 80 (74.1) | ns | 132 (73.3) | 67 (88.2) | 0.009 |
| Clinical-Pathological Correlation Analysis Ability | 206 (80.5) | 88 (79.3) | 118 (81.4) | ns | 143 (79.4) | 63 (82.9) | ns | 113 (74.8) | 93 (88.6) | 0.006 | 145 (77.1) | 61 (89.7) | 0.031 | 118 (79.7) | 88 (81.5) | ns | 141 (78.3) | 65 (85.5) | ns |
| Confidence in Diagnosis and Treatment of Complex Cases | 157 (61.3) | 89 (80.2) | 68 (46.9) | ＜0.001 | 110 (61.1) | 47 (61.8) | ns | 84 (55.6) | 73 (69.5) | 0.027 | 108 (57.4) | 49 (72.1) | 0.042 | 84 (56.8) | 73 (67.6) | ns | 104 (57.8) | 53 (69.7) | 0.092 |
| Communication Efficiency with Pathologists | 129 (50.4) | 57 (51.4) | 72 (49.7) | ns | 86 (47.8) | 43 (56.6) | ns | 68 (45.0) | 61 (58.1) | 0.043 | 89 (47.3) | 40 (58.8) | 0.12 | 71 (48.0) | 58 (53.7) | ns | 87 (48.3) | 42 (55.3) | ns |
| No Significant Improvement Perceived | 33 (12.9) | 18 (16.2) | 15 (10.3) | ns | 23 (12.8) | 10 (13.2) | ns | 20 (13.2) | 13 (12.4) | ns | 23 (12.2) | 10 (14.7) | ns | 22 (14.9) | 11 (10.2) | ns | 26 (14.4) | 7 (9.2) | ns |
| What are the main difficulties you face in participating in renal pathology continuing medical education? | | | | | | | | | | | | | | | | | | | |
| Time Conflict (Busy Clinical Work, Difficulty in Sparing Time) | 195 (76.2) | 81 (73.0) | 114 (78.6) | ns | 136 (75.6) | 59 (77.6) | ns | 113 (74.8) | 82 (78.1) | ns | 140 (74.5) | 55 (80.9) | ns | 115 (77.7) | 80 (74.1) | ns | 137 (76.1) | 58 (76.3) | ns |
| Training Resources Concentrated in Large Cities, Inconvenient for Grassroots Participation | 178 (69.5) | 81 (73.0) | 97 (66.9) | ns | 129 (71.7) | 49 (64.5) | ns | 102 (67.5) | 76 (72.4) | ns | 129 (68.6) | 49 (72.1) | ns | 94 (63.5) | 84 (77.8) | 0.019 | 132 (73.3) | 46 (60.5) | 0.053 |
| Mismatch Between Training Content and Personal Needs (e.g., Tertiary Hospital Physicians Find Content Too Basic; Grassroots Physicians Find It Too Complex) | 171 (66.8) | 71 (64.0) | 100 (69.0) | ns | 122 (67.8) | 49 (64.5) | ns | 96 (63.6) | 75 (71.4) | ns | 124 (66.0) | 47 (69.1) | ns | 93 (62.8) | 78 (72.2) | ns | 125 (69.4) | 46 (60.5) | ns |
| Lack of Funding Support (e.g., Training Fees, Travel Expenses) | 127 (49.6) | 58 (52.3) | 69 (47.6) | ns | 86 (47.8) | 41 (53.9) | ns | 64 (42.4) | 63 (60.0) | 0.007 | 85 (45.2) | 42 (61.8) | 0.023 | 68 (45.9) | 59 (54.6) | ns | 89 (49.4) | 38 (50.0) | ns |
| Single Training Format (e.g., Online Only, Lack of Practical Operation) | 88 (34.4) | 41 (36.9) | 47 (32.4) | ns | 60 (33.3) | 28 (36.8) | ns | 48 (31.8) | 40 (38.1) | ns | 63 (33.5) | 25 (36.8) | ns | 48 (32.4) | 40 (37.0) | ns | 59 (32.8) | 29 (38.2) | ns |
| What do you think are the main problems in current renal pathology continuing medical education? | | | | | | | | | | | | | | | | | | | |
| Low Training Frequency (e.g., Only 1–2 Times/Year) | 183 (71.5) | 82 (73.9) | 101 (69.7) | ns | 124 (68.9) | 59 (77.6) | ns | 103 (68.2) | 80 (76.2) | ns | 133 (70.7) | 50 (73.5) | ns | 101 (68.2) | 82 (75.9) | ns | 131 (72.8) | 52 (68.4) | ns |
| Disconnection Between Content and Clinical Practice (e.g., Pure Theory, Lack of Case Analysis) | 166 (64.8) | 75 (67.6) | 91 (62.8) | ns | 118 (65.6) | 48 (63.2) | ns | 92 (60.9) | 74 (70.5) | 0.143 | 117 (62.2) | 49 (72.1) | ns | 89 (60.1) | 77 (71.3) | ns | 121 (67.2) | 45 (59.2) | ns |
| Single Format (e.g., Offline Lectures Only, No Online Playback) | 164 (64.1) | 68 (61.3) | 96 (66.2) | ns | 118 (65.6) | 46 (60.5) | ns | 89 (58.9) | 75 (71.4) | 0.047 | 119 (63.3) | 45 (66.2) | ns | 95 (64.2) | 69 (63.9) | ns | 118 (65.6) | 46 (60.5) | ns |
| Lack of Targeting (e.g., Failing to Distinguish Needs of Physicians with Different Titles) | 142 (55.5) | 55 (49.5) | 87 (60.0) | 0.101 | 96 (53.3) | 46 (60.5) | ns | 72 (47.7) | 70 (66.7) | 0.003 | 97 (51.6) | 45 (66.2) | 0.046 | 81 (54.7) | 61 (56.5) | ns | 101 (56.1) | 41 (53.9) | ns |
| Absence of Assessment Mechanism (No Feedback on Training Effects After Completion) | 74 (28.9) | 33 (29.7) | 41 (28.3) | ns | 52 (28.9) | 22 (28.9) | ns | 37 (24.5) | 37 (35.2) | ns | 51 (27.1) | 23 (33.8) | ns | 41 (27.7) | 33 (30.6) | ns | 52 (28.9) | 22 (28.9) | ns |
| ns，not significant; | | | | | | | | | | | | | | | | | | | |
