# Supplementary material for: Continuing medical education in renal pathology: current practices and needs among nephrologists
Source: BMC Med Educ. 2026 Feb 12;26:441. doi: 10.1186/s12909-026-08798-4 (PMC12997942; doi:10.1186/s12909-026-08798-4)
Supplement: Supplementary file 4 — Supplementary Material 4. [file 12909_2026_8798_MOESM4_ESM.docx]

**Supplemental Table 3. Analysis of Questionnaire Results on the Status of Primary Medical Institutions, Third-Party Institutions and AI in Renal Pathology Continuing Medical Education**

|  | All (n=256) | Sex | | | Age | | | Working time | | | Medical Professional Title | | | Affiliation | | | Report Interpretation Volume | | |
| --- | --- | --- | --- | --- | --- | --- | --- | --- | --- | --- | --- | --- | --- | --- | --- | --- | --- | --- | --- |
|  |  | Male ( n=111） | Female (n=145) | p | ≤40y（n=180） | ＞40y（n=76） | p | ≤10 year（n=151） | ＞10year（n=105） | p | Attending Physician and Below（n=188） | Associate Chief Physician and Above（n=68） | p | Grade A Tertiary Hospital（n=148） | Grade B Tertiary Hospital and Below（n=108） | p | ≤10cases/year（n=180） | ＞10cases/year（n=76） | p |
| Basic Situation of Primary Medical Institutions, Third-Party Institutions and AI | | | | | | | | | | | | | | | | | | | |
| What do you think are the core needs of primary medical institutions for renal pathology? | | | | | | | | | | | | | | | | | | | |
| Channels for rapid specimen transportation to external institutions | 196 (76.6) | 82 (73.9) | 114 (78.6) | ns | 143 (79.4) | 53 (69.7) | ns | 118 (78.1) | 78 (74.3) | ns | 145 (77.1) | 51 (75.0) | ns | 114 (77.0) | 82 (75.9) | ns | 138 (76.7) | 58 (76.3) | ns |
| Simplified pathology report | 159 (62.1) | 69 (62.2) | 90 (62.1) | ns | 115 (63.9) | 44 (57.9) | ns | 91 (60.3) | 68 (64.8) | ns | 115 (61.2) | 44 (64.7) | ns | 85 (57.4) | 74 (68.5) | ns | 116 (64.4) | 43 (56.6) | ns |
| Telepathology consultation support | 186 (72.7) | 77 (69.4) | 109 (75.2) | ns | 129 (71.7) | 57 (75.0) | ns | 101 (66.9) | 85 (81.0) | 0.015 | 132 (70.2) | 54 (79.4) | ns | 106 (71.6) | 80 (74.1) | ns | 129 (71.7) | 57 (75.0) | ns |
| Renal pathology knowledge training tailored for primary institutions | 177 (69.1) | 66 (59.5) | 111 (76.6) | 0.004 | 122 (67.8) | 55 (72.4) | ns | 95 (62.9) | 82 (78.1) | 0.013 | 123 (65.4) | 54 (79.4) | ns | 103 (69.6) | 74 (68.5) | ns | 126 (70.0) | 51 (67.1) | ns |
| What are the main restrictive factors for primary institutions to carry out renal pathology-related work? | | | | | | | | | | | | | | | | | | | |
| Lack of specimen processing equipment | 195 (76.2) | 81 (73.0) | 114 (78.6) | ns | 143 (79.4) | 52 (68.4) | ns | 119 (78.8) | 76 (72.4) | ns | 150 (79.8) | 45 (66.2) | 0.031 | 106 (71.6) | 89 (82.4) | ns | 139 (77.2) | 56 (73.7) | ns |
| Insufficient technical personnel | 209 (81.6) | 88 (79.3) | 121 (83.4) | ns | 145 (80.6) | 64 (84.2) | ns | 116 (76.8) | 93 (88.6) | ns | 150 (79.8) | 59 (86.8) | ns | 115 (77.7) | 94 (87.0) | ns | 150 (83.3) | 59 (77.6) | ns |
| High cost of external specimen transportation | 142 (55.5) | 55 (49.5) | 87 (60.0) | ns | 106 (58.9) | 36 (47.4) | 0.1 | 81 (53.6) | 61 (58.1) | ns | 108 (57.4) | 34 (50.0) | ns | 77 (52.0) | 65 (60.2) | ns | 100 (55.6) | 42 (55.3) | ns |
| Unsmooth cooperation mechanism with pathology departments of superior hospitals | 147 (57.4) | 60 (54.1) | 87 (60.0) | ns | 100 (55.6) | 47 (61.8) | ns | 76 (50.3) | 71 (67.6) | 0.007 | 104 (55.3) | 43 (63.2) | ns | 92 (62.2) | 55 (50.9) | 0.075 | 104 (57.8) | 43 (56.6) | ns |
| Does your institution rely on third-party pathological testing institutions? | | | | | | | | | | | | | | | | | | | |
| Yes (regularly send to professional pathological testing companies) | 107 (41.8) | 58 (52.3) | 49 (33.8) | 0.003 | 66 (36.7) | 41 (53.9) | 0.013 | 60 (39.7) | 47 (44.8) | ns | 67 (35.6) | 40 (58.8) | 0.001 | 64 (43.2) | 43 (39.8) | ns | 69 (38.3) | 38 (50.0) | ns |
| Yes (regularly send to superior hospitals, e.g., primary hospitals send to tertiary hospitals) | 72 (28.1) | 31 (27.9) | 41 (28.3) | ns | 54 (30.0) | 18 (23.7) | ns | 49 (32.5) | 23 (21.9) | ns | 57 (30.3) | 15 (22.1) | ns | 39 (26.4) | 33 (30.6) | ns | 50 (27.8) | 22 (28.9) | ns |
| No (with its own pathology department) | 58 (22.7) | 13 (11.7) | 45 (31.0) | ＜0.001 | 47 (26.1) | 11 (14.5) | 0.05 | 42 (27.8) | 16 (15.2) | ns | 47 (25.0) | 11 (16.2) | ns | 44 (29.7) | 14 (13.0) | 0.001 | 43 (23.9) | 15 (19.7) | ns |
| What does your trust in third-party institutions mainly depend on? | | | | | | | | | | | | | | | | | | | |
| Testing qualifications (e.g., certified laboratories) | 219 (85.5) | 90 (81.1) | 129 (89.0) | ns | 156 (86.7) | 63 (82.9) | ns | 130 (86.1) | 89 (84.8) | ns | 163 (86.7) | 56 (82.4) | ns | 128 (86.5) | 91 (84.3) | ns | 155 (86.1) | 64 (84.2) | ns |
| Timeliness of reports | 164 (64.1) | 73 (65.8) | 91 (62.8) | ns | 116 (64.4) | 48 (63.2) | ns | 87 (57.6) | 77 (73.3) | 0.012 | 115 (61.2) | 49 (72.1) | ns | 96 (64.9) | 68 (63.0) | ns | 120 (66.7) | 44 (57.9) | ns |
| Ability to provide clinical interpretation suggestions | 161 (62.9) | 69 (62.2) | 92 (63.4) | ns | 112 (62.2) | 49 (64.5) | ns | 81 (53.6) | 80 (76.2) | <0.001 | 109 (58.0) | 52 (76.5) | 0.008 | 96 (64.9) | 65 (60.2) | ns | 113 (62.8) | 48 (63.2) | ns |
| Reasonableness of prices | 105 (41.0) | 46 (41.4) | 59 (40.7) | ns | 74 (41.1) | 31 (40.8) | ns | 53 (35.1) | 52 (49.5) | 0.028 | 74 (39.4) | 31 (45.6) | ns | 58 (39.2) | 47 (43.5) | ns | 78 (43.3) | 27 (35.5) | ns |
| What do you think is the potential value of AI in renal pathology? | | | | | | | | | | | | | | | | | | | |
| Rapid preliminary screening (e.g., identifying typical lesions, reducing manual workload) | 224 (87.5) | 93 (83.8) | 131 (90.3) | ns | 157 (87.2) | 67 (88.2) | ns | 130 (86.1) | 94 (89.5) | ns | 164 (87.2) | 60 (88.2) | ns | 130 (87.8) | 94 (87.0) | ns | 158 (87.8) | 66 (86.8) | ns |
| Quantitative analysis (e.g., automatic counting of glomerulosclerosis ratio) | 195 (76.2) | 86 (77.5) | 109 (75.2) | ns | 136 (75.6) | 59 (77.6) | ns | 109 (72.2) | 86 (81.9) | ns | 139 (73.9) | 56 (82.4) | ns | 113 (76.4) | 82 (75.9) | ns | 136 (75.6) | 59 (77.6) | ns |
| Assisting primary physicians in report interpretation (e.g., AI generates popularized conclusions) | 180 (70.3) | 73 (65.8) | 107 (73.8) | ns | 127 (70.6) | 53 (69.7) | ns | 98 (64.9) | 82 (78.1) | 0.026 | 126 (67.0) | 54 (79.4) | 0.064 | 109 (73.6) | 71 (65.7) | ns | 128 (71.1) | 52 (68.4) | ns |
| No significant value (relying on pathologists' experience is more reliable) | 29 (11.3) | 16 (14.4) | 13 (9.0) | ns | 18 (10.0) | 11 (14.5) | ns | 15 (9.9) | 14 (13.3) | ns | 21 (11.2) | 8 (11.8) | ns | 20 (13.5) | 9 (8.3) | ns | 21 (11.7) | 8 (10.5) | ns |
| What are your main concerns about the application of AI? |  |  |  |  |  |  |  |  |  |  |  |  |  |  |  |  |  |  |  |
| Misdiagnosis risk (especially for rare/complex cases) | 205 (80.1) | 86 (77.5) | 119 (82.1) | ns | 145 (80.6) | 60 (78.9) | ns | 121 (80.1) | 84 (80.0) | ns | 155 (82.4) | 50 (73.5) | ns | 123 (83.1) | 82 (75.9) | ns | 143 (79.4) | 62 (81.6) | ns |
| Data privacy leakage (patients' pathological images) | 161 (62.9) | 67 (60.4) | 94 (64.8) | ns | 114 (63.3) | 47 (61.8) | ns | 91 (60.3) | 70 (66.7) | ns | 115 (61.2) | 46 (67.6) | ns | 90 (60.8) | 71 (65.7) | ns | 114 (63.3) | 47 (61.8) | ns |
| Over-reliance on AI, weakening physicians' subjective judgment ability | 183 (71.5) | 71 (64.0) | 112 (77.2) | 0.025 | 125 (69.4) | 58 (76.3) | ns | 93 (61.6) | 90 (85.7) | <0.001 | 131 (69.7) | 52 (76.5) | ns | 101 (68.2) | 82 (75.9) | ns | 132 (73.3) | 51 (67.1) | ns |
| Lack of unified standards, inconsistent results among different AI systems | 153 (59.8) | 59 (53.2) | 94 (64.8) | ns | 104 (57.8) | 49 (64.5) | ns | 82 (54.3) | 71 (67.6) | 0.038 | 106 (56.4) | 47 (69.1) | 0.083 | 91 (61.5) | 62 (57.4) | ns | 103 (57.2) | 50 (65.8) | ns |
| ns, not significant; | | | | | | | | | | | | | | | | | | | |
