# Supplementary material for: Continuing medical education in renal pathology: current practices and needs among nephrologists
Source: BMC Med Educ. 2026 Feb 12;26:441. doi: 10.1186/s12909-026-08798-4 (PMC12997942; doi:10.1186/s12909-026-08798-4)
Supplement: Supplementary file 5 — Supplementary Material 5. [file 12909_2026_8798_MOESM5_ESM.docx]

**Supplemental Table 4. Analysis of Questionnaire Results on the Importance, Methods and Approaches of Continuing Medical Education**

|  | **All (n=256)** | **Sex** | | | **Age** | | | **Working time** | | | **Medical Professional Title** | | | Affiliation | | | **Report Interpretation Volume** | | |
| --- | --- | --- | --- | --- | --- | --- | --- | --- | --- | --- | --- | --- | --- | --- | --- | --- | --- | --- | --- |
|  |  | **Male ( n=111）** | **Female (n=145)** | **p** | **≤40y（n=180）** | **＞40y（n=76）** | p | **≤10 year（n=151）** | **＞10year（n=105）** | **p** | **Attending Physician and Below（n=188）** | **Associate Chief Physician and Above（n=68）** | **p** | **Grade A Tertiary Hospital（n=148）** | **Grade B Tertiary Hospital and Below（n=108）** | **p** | **≤10cases/year（n=180）** | **＞10cases/year（n=76）** | **p** |
| **Current Status, Problems and Difficulties of Renal Pathology-related Training** | | | | | | | | | | | | | | | | | | | |
| What Channels Do You Mainly Use to Learn and Update Renal Pathology Knowledge? | | | | | | | | | | | | | | | | | | | |
| Medical School Courses | 149 (58.2) | **61** (55.0) | 88 (60.7) | **ns** | 113 (62.8) | 36 (47.4) | **0.027** | 93 (61.6) | 56 (53.3) | **ns** | 117 (62.2) | 32 (47.1) | **0.032** | 85 (57.4) | 64 (59.3) | ns | 106 (58.9) | 43 (56.6) | **ns** |
| **Resident/Specialist Standardized Training** | 145 (56.6) | **59** (53.2) | 86 (59.3) | **ns** | 117 (65.0) | 28 (36.8) | **＜0.001** | 99 (65.6) | 46 (43.8) | **0.001** | 120 (63.8) | 25 (36.8) | **<0.001** | 76 (51.4) | 69 (63.9) | 0.055 | 100 (55.6) | 45 (59.2) | **ns** |
| Attending Academic Conferences/Lectures/Training Courses | 196 (76.6) | **87** (78.4) | 109 (75.2) | **ns** | 134 (74.4) | 62 (81.6) | **ns** | 103 (68.2) | 93 (88.6) | **<0.001** | 137 (72.9) | 59 (86.8) | **ns** | 110 (74.3) | 86 (79.6) | ns | 134 (74.4) | 62 (81.6) | **ns** |
| Reading Professional Books and Journal Literature | 159 (62.1) | **65** (58.6) | 94 (64.8) | **ns** | 110 (61.1) | 49 (64.5) | **ns** | 81 (53.6) | 78 (74.3) | **0.001** | 111 (59.0) | 48 (70.6) | **0.109** | 90 (60.8) | 69 (63.9) | ns | 102 (56.7) | 57 (75.0) | **0.007** |
| Online Learning Resources | 156 (60.9) | **71** (64.0) | 85 (58.6) | **ns** | 112 (62.2) | 44 (57.9) | **ns** | 84 (55.6) | 72 (68.6) | **0.038** | 109 (58.0) | 47 (69.1) | **0.113** | 86 (58.1) | 70 (64.8) | ns | 102 (56.7) | 54 (71.1) | **0.036** |
| Communication with Pathologists/Joint Slide Review | 104 (40.6) | **43** (38.7) | 61 (42.1) | **ns** | 66 (36.7) | 38 (50.0) | **0.052** | 52 (34.4) | 52 (49.5) | **0.02** | 69 (36.7) | 35 (51.5) | **0.043** | 60 (40.5) | 44 (40.7) | ns | 64 (35.6) | 40 (52.6) | **0.012** |
| Case Conferences (Departmental/MDT) | 106 (41.4) | **45** (40.5) | 61 (42.1) | **ns** | 69 (38.3) | 37 (48.7) | **0.129** | 53 (35.1) | 53 (50.5) | **0.015** | 71 (37.8) | 35 (51.5) | **0.062** | 62 (41.9) | 44 (40.7) | ns | 69 (38.3) | 37 (48.7) | **ns** |
| Self-study | 75 (29.3) | **37** (33.3) | 38 (26.2) | **ns** | 52 (28.9) | 23 (30.3) | **ns** | 39 (25.8) | 36 (34.3) | **ns** | 55 (29.3) | 20 (29.4) | **ns** | 41 (27.7) | 34 (31.5) | ns | 52 (28.9) | 23 (30.3) | **ns** |
| **Do You Currently Participate in Renal Pathology-related Training (e.g., Academic Conferences, Pathology Slide Seminars)?** | | | | | | | | | | | | | | | | | | | |
| ≥ 1 Time/Year | 136 (53.1) | **65** (58.6) | 71 (49.0) | 0.132 | 99 (55.0) | 37 (48.7) | **ns** | 91 (60.3) | 45 (42.9) | **0.007** | 105 (55.9) | 31 (45.6) | **ns** | 84 (56.8) | 52 (48.1) | ns | 82 (45.6) | 54 (71.1) | **<0.001** |
| Do You Think the Following Abilities Have Improved After Attending Renal Pathology-related Training? | | | | | | | | | | | | | | | | | | | |
| Accuracy of Pathology Report Interpretation | 199 (77.7) | **82** (73.9) | 117 (80.7) | **ns** | 138 (76.7) | 61 (80.3) | **ns** | 112 (74.2) | 87 (82.9) | **ns** | 144 (76.6) | 55 (80.9) | **ns** | 119 (80.4) | 80 (74.1) | ns | 132 (73.3) | 67 (88.2) | **0.009** |
| Clinical-Pathological Correlation Analysis Ability | 206 (80.5) | **88** (79.3) | 118 (81.4) | **ns** | 143 (79.4) | 63 (82.9) | **ns** | 113 (74.8) | 93 (88.6) | **0.006** | 145 (77.1) | 61 (89.7) | **0.031** | 118 (79.7) | 88 (81.5) | ns | 141 (78.3) | 65 (85.5) | **ns** |
| Confidence in Diagnosis and Treatment of Complex Cases | 157 (61.3) | **89** (80.2) | 68 (46.9) | **＜0.001** | 110 (61.1) | 47 (61.8) | **ns** | 84 (55.6) | 73 (69.5) | **0.027** | 108 (57.4) | 49 (72.1) | **0.042** | 84 (56.8) | 73 (67.6) | ns | 104 (57.8) | 53 (69.7) | **0.092** |
| **Efficiency of Communication with Pathologists** | 129 (50.4) | **57** (51.4) | 72 (49.7) | **ns** | 86 (47.8) | 43 (56.6) | **ns** | 68 (45.0) | 61 (58.1) | **0.043** | 89 (47.3) | 40 (58.8) | **0.12** | 71 (48.0) | 58 (53.7) | ns | 87 (48.3) | 42 (55.3) | **ns** |
| No Significant Improvement Perceived | 33 (12.9) | **18** (16.2) | 15 (10.3) | **ns** | 23 (12.8) | 10 (13.2) | **ns** | 20 (13.2) | 13 (12.4) | **ns** | 23 (12.2) | 10 (14.7) | **ns** | 22 (14.9) | 11 (10.2) | ns | 26 (14.4) | 7 (9.2) | **ns** |
| **What Are the Main Difficulties You Face in Participating in Renal Pathology Continuing Education?** | | | | | | | | | | | | | | | | | | | |
| Time Conflict (Busy Clinical Work, Difficulty in Spare Time) | 195 (76.2) | **81** (73.0) | 114 (78.6) | **ns** | 136 (75.6) | 59 (77.6) | **ns** | 113 (74.8) | 82 (78.1) | **ns** | 140 (74.5) | 55 (80.9) | **ns** | 115 (77.7) | 80 (74.1) | ns | 137 (76.1) | 58 (76.3) | **ns** |
| Training Resources Concentrated in Large Cities, Inconvenient for Grassroots Participation | 178 (69.5) | **81** (73.0) | 97 (66.9) | **ns** | 129 (71.7) | 49 (64.5) | **ns** | 102 (67.5) | 76 (72.4) | **ns** | 129 (68.6) | 49 (72.1) | **ns** | 94 (63.5) | 84 (77.8) | 0.019 | 132 (73.3) | 46 (60.5) | **0.053** |
| **Mismatch Between Training Content and Personal Needs** | 171 (66.8) | **71** (64.0) | 100 (69.0) | **ns** | 122 (67.8) | 49 (64.5) | **ns** | 96 (63.6) | 75 (71.4) | **ns** | 124 (66.0) | 47 (69.1) | **ns** | 93 (62.8) | 78 (72.2) | ns | 125 (69.4) | 46 (60.5) | **ns** |
| Lack of Funding Support | 127 (49.6) | **58** (52.3) | 69 (47.6) | **ns** | 86 (47.8) | 41 (53.9) | **ns** | 64 (42.4) | 63 (60.0) | **0.007** | 85 (45.2) | 42 (61.8) | **0.023** | 68 (45.9) | 59 (54.6) | ns | 89 (49.4) | 38 (50.0) | **ns** |
| Single Training Format | 88 (34.4) | **41** (36.9) | 47 (32.4) | **ns** | 60 (33.3) | 28 (36.8) | **ns** | 48 (31.8) | 40 (38.1) | **ns** | 63 (33.5) | 25 (36.8) | **ns** | 48 (32.4) | 40 (37.0) | ns | 59 (32.8) | 29 (38.2) | **ns** |
| **What Do You Think Are the Main Problems in Current Renal Pathology Continuing Medical Education?** | | | | | | | | | | | | | | | | | | | |
| Low Training Frequency | 183 (71.5) | **82** (73.9) | 101 (69.7) | **ns** | 124 (68.9) | 59 (77.6) | **ns** | 103 (68.2) | 80 (76.2) | **ns** | 133 (70.7) | 50 (73.5) | **ns** | 101 (68.2) | 82 (75.9) | ns | 131 (72.8) | 52 (68.4) | **ns** |
| Disconnection Between Content and Clinical Practice (e.g., Pure Theory, Lack of Case Analysis) | 166 (64.8) | **75** (67.6) | 91 (62.8) | **ns** | 118 (65.6) | 48 (63.2) | **ns** | 92 (60.9) | 74 (70.5) | **0.143** | 117 (62.2) | 49 (72.1) | **ns** | 89 (60.1) | 77 (71.3) | ns | 121 (67.2) | 45 (59.2) | **ns** |
| Single Format | 164 (64.1) | **68** (61.3) | 96 (66.2) | **ns** | 118 (65.6) | 46 (60.5) | **ns** | 89 (58.9) | 75 (71.4) | **0.047** | 119 (63.3) | 45 (66.2) | **ns** | 95 (64.2) | 69 (63.9) | ns | 118 (65.6) | 46 (60.5) | **ns** |
| Lack of Targetedness | 142 (55.5) | **55** (49.5) | 87 (60.0) | 0.101 | 96 (53.3) | 46 (60.5) | **ns** | 72 (47.7) | 70 (66.7) | **0.003** | 97 (51.6) | 45 (66.2) | **0.046** | 81 (54.7) | 61 (56.5) | ns | 101 (56.1) | 41 (53.9) | **ns** |
| Lack of Assessment Mechanism (No Effect Feedback After Training) | 74 (28.9) | **33** (29.7) | 41 (28.3) | **ns** | 52 (28.9) | 22 (28.9) | **ns** | 37 (24.5) | 37 (35.2) | **ns** | 51 (27.1) | 23 (33.8) | **ns** | 41 (27.7) | 33 (30.6) | ns | 52 (28.9) | 22 (28.9) | **ns** |
| **Importance, Methods and Approaches of Continuing Medical Education** | | | | | | | | | | | | | | | | | | | |
| **What Renal Pathology Training Content Do You Think Needs the Most Strengthening?** | | | | | | | | | | | | | | | | | | | |
| Pathological Features of Common Renal Diseases | 224 (87.5) | **95** (85.6) | 129 (89.0) | **ns** | 158 (87.8) | 66 (86.8) | **ns** | 132 (87.4) | 92 (87.6) | **ns** | 161 (85.6) | 63 (92.6) | **ns** | 128 (86.5) | 96 (88.9) | ns | 155 (86.1) | 69 (90.8) | **ns** |
| Systematic Interpretation Methods of Pathology Reports | 215 (84.0) | **92** (82.9) | 123 (84.8) | **ns** | 151 (83.9) | 64 (84.2) | **ns** | 121 (80.1) | 94 (89.5) | **ns** | 155 (82.4) | 60 (88.2) | **ns** | 125 (84.5) | 90 (83.3) | ns | 151 (83.9) | 64 (84.2) | **ns** |
| Clinical-Pathological Case Analysis | 200 (78.1) | **82** (73.9) | 118 (81.4) | **ns** | 141 (78.3) | 59 (77.6) | **ns** | 110 (72.8) | 90 (85.7) | **0.014** | 142 (75.5) | 58 (85.3) | **ns** | 114 (77.0) | 86 (79.6) | ns | 141 (78.3) | 59 (77.6) | **ns** |
| Application of New Renal Pathology Technologies | 160 (62.5) | **64** (57.7) | 96 (66.2) | **ns** | 105 (58.3) | 55 (72.4) | **0.035** | 83 (55.0) | 77 (73.3) | **0.004** | 110 (58.5) | 50 (73.5) | **0.029** | 90 (60.8) | 70 (64.8) | ns | 111 (61.7) | 49 (64.5) | **ns** |
| **What Do You Think Is the Most Effective Training Format?** | | | | | | | | | | | | | | | | | | | |
| Clinical-Pathological Case Conferences | 222 (86.7) | **92** (82.9) | 130 (89.7) | **ns** | 154 (85.6) | 68 (89.5) | **ns** | 130 (86.1) | 92 (87.6) | **ns** | 163 (86.7) | 59 (86.8) | **ns** | 130 (87.8) | 92 (85.2) | ns | 153 (85.0) | 69 (90.8) | **ns** |
| Practical Pathology Slide Review | 199 (77.7) | **86** (77.5) | 113 (77.9) | **ns** | 142 (78.9) | 57 (75.0) | **ns** | 113 (74.8) | 86 (81.9) | **ns** | 145 (77.1) | 54 (79.4) | **ns** | 118 (79.7) | 81 (75.0) | ns | 139 (77.2) | 60 (78.9) | **ns** |
| Online Recorded Courses | 161 (62.9) | **69** (62.2) | 92 (63.4) | **ns** | 115 (63.9) | 46 (60.5) | **ns** | 89 (58.9) | 72 (68.6) | **ns** | 115 (61.2) | 46 (67.6) | **ns** | 90 (60.8) | 71 (65.7) | ns | 114 (63.3) | 47 (61.8) | **ns** |
| Face-to-face Q&A with Experts | 148 (57.8) | **56** (50.5) | 92 (63.4) | **0.042** | 106 (58.9) | 42 (55.3) | **ns** | 79 (52.3) | 69 (65.7) | **0.04** | 109 (58.0) | 39 (57.4) | **ns** | 84 (56.8) | 64 (59.3) | ns | 102 (56.7) | 46 (60.5) | **ns** |
| Skill Assessment | 86 (33.6) | **39** (35.1) | 47 (32.4) | **ns** | 61 (33.9) | 25 (32.9) | **ns** | 43 (28.5) | 43 (41.0) | **0.044** | 61 (32.4) | 25 (36.8) | **ns** | 46 (31.1) | 40 (37.0) | ns | 61 (33.9) | 25 (32.9) | **ns** |
| **What Training Cycle Do You Expect for Renal Pathology-related Programs?** | | | | | | | | | | | | | | | | | | | |
| Once a Month | 85 (33.2) | **34** (30.6) | 51 (35.2) | **ns** | 57 (31.7) | 28 (36.8) | **ns** | 47 (31.1) | 38 (36.2) | **ns** | 63 (33.5) | 22 (32.4) | **ns** | 52 (35.1) | 33 (30.6) | ns | 57 (31.7) | 28 (36.8) | **ns** |
| Once a Quarter | 85 (33.2) | **41** (36.9) | 44 (30.3) | **ns** | 66 (36.7) | 19 (25.0) | **0.082** | 55 (36.4) | 30 (28.6) | **ns** | 62 (33.0) | 23 (33.8) | **ns** | 54 (36.5) | 31 (28.7) | ns | 56 (31.1) | 29 (38.2) | **ns** |
| Once Every 6 Months / 1-2 Times a Year | 62 (24.2) | **27** (24.3) | 35 (24.1) | **ns** | 38 (21.1) | 24 (31.6) | **0.081** | 32 (21.2) | 30 (28.6) | **ns** | 43 (22.9) | 19 (27.9) | **ns** | 31 (20.9) | 31 (28.7) | ns | 48 (26.7) | 14 (18.4) | **ns** |
| On-demand Implementation | 24 (9.4) | **9** (8.1) | 15 (10.3) | **ns** | 19 (10.6) | 5 (6.6) | **ns** | 17 (11.3) | 7 (6.7) | **ns** | 20 (10.6) | 4 (5.9) | **ns** | 13 (8.8) | 11 (10.2) | ns | 19 (10.6) | 5 (6.6) | **ns** |
| **In Which Aspects Do You Most Want to Obtain More Renal Pathology Training or Resources?** | | | | | | | | | | | | | | | | | | | |
| Key Points and Pitfalls of Pathological Diagnosis for Common and Rare Renal Diseases | 207 (80.9) | **81** (73.0) | 126 (86.9) | **0.006** | 140 (77.8) | 67 (88.2) | **ns** | 115 (76.2) | 92 (87.6) | **0.024** | 148 (78.7) | 59 (86.8) | **ns** | 125 (84.5) | 82 (75.9) | ns | 141 (78.3) | 66 (86.8) | **ns** |
| In-depth Interpretation and Clinical Significance of Renal Biopsy Reports | 204 (79.7) | **88** (79.3) | 116 (80.0) | **ns** | 148 (82.2) | 56 (73.7) | **ns** | 115 (76.2) | 89 (84.8) | **ns** | 146 (77.7) | 58 (85.3) | **ns** | 118 (79.7) | 86 (79.6) | ns | 142 (78.9) | 62 (81.6) | **ns** |
| Detailed Explanation and Application of Pathological Classification/Scoring Systems | 167 (65.2) | **69** (62.2) | 98 (67.6) | **ns** | 115 (63.9) | 52 (68.4) | **ns** | 90 (59.6) | 77 (73.3) | **0.024** | 116 (61.7) | 51 (75.0) | **0.054** | 91 (61.5) | 76 (70.4) | ns | 114 (63.3) | 53 (69.7) | **ns** |
| Interpretation Skills of Light Microscopy, Immunofluorescence and Electron Microscopy Images | 175 (68.4) | **71** (64.0) | 104 (71.7) | **ns** | 125 (69.4) | 50 (65.8) | **ns** | 97 (64.2) | 78 (74.3) | **ns** | 125 (66.5) | 50 (73.5) | **ns** | 104 (70.3) | 71 (65.7) | ns | 125 (69.4) | 50 (65.8) | **ns** |
| Integration of Latest Clinical Guidelines and Renal Pathology Knowledge | 158 (61.7) | **62** (55.9) | 96 (66.2) | **ns** | 114 (63.3) | 44 (57.9) | **ns** | 86 (57.0) | 72 (68.6) | **0.068** | 114 (60.6) | 44 (64.7) | **ns** | 100 (67.6) | 58 (53.7) | 0.027 | 108 (60.0) | 50 (65.8) | **ns** |
| How to Better Communicate and Collaborate with Pathologists | 101 (39.5) | **41** (36.9) | 60 (41.4) | **ns** | 71 (39.4) | 30 (39.5) | **ns** | 52 (34.4) | 49 (46.7) | **0.052** | 75 (39.5) | 31 (45.6) | **ns** | 61 (41.2) | 40 (37.0) | ns | 68 (37.8) | 33 (43.4) | **ns** |
| Latest Research Progress in Renal Pathology | 121 (47.3) | **43** (38.7) | 78 (53.8) | **0.023** | 80 (44.4) | 41 (53.9) | **ns** | 63 (41.7) | 58 (55.2) | **0.042** | 83 (44.1) | 38 (55.9) | **0.119** | 78 (52.7) | 43 (39.8) | 0.044 | 83 (46.1) | 38 (50.0) | **ns** |
| Recommendations for Online Pathology Atlases/Database Resources | 95 (37.1) | **39** (35.1) | 56 (38.6) | **ns** | 65 (36.1) | 30 (39.5) | **ns** | 50 (33.1) | 45 (42.9) | **ns** | 68 (36.2) | 27 (39.7) | **ns** | 62 (41.9) | 33 (30.6) | ns | 65 (36.1) | 30 (39.5) | **ns** |
| Practical Slide Review Workshops | 85 (33.2) | **37** (33.3) | 48 (33.1) | **ns** | 58 (32.2) | 27 (35.5) | **ns** | 46 (30.5) | 39 (37.1) | **ns** | 59 (31.4) | 26 (38.2) | **ns** | 50 (33.8) | 35 (32.4) | ns | 61 (33.9) | 24 (31.6) | **ns** |
| **What Impacts Do You Think Systematic Renal Pathology Continuing Education May Have on Patient Outcomes?** | | | | | | | | | | | | | | | | | | | |
| Reduce Misdiagnosis Rate and Unnecessary Treatment | 211 (82.4) | **84** (75.7) | 127 (87.6) | **0.02** | 142 (78.9) | 69 (90.8) | **0.03** | 119 (78.8) | 92 (87.6) | **ns** | 153 (81.4) | 58 (85.3) | **ns** | 120 (81.1) | 91 (84.3) | ns | 150 (83.3) | 61 (80.3) | **ns** |
| Improve Matching Degree Between Treatment Plans and Pathological Types, Enhance Therapeutic Effect | 213 (83.2) | **93** (83.8) | 120 (82.8) | **ns** | 149 (82.8) | 64 (84.2) | **ns** | 118 (78.1) | 95 (90.5) | **0.011** | 149 (79.3) | 64 (94.1) | **0.004** | 123 (83.1) | 90 (83.3) | ns | 151 (83.9) | 62 (81.6) | **ns** |
| Shorten Diagnosis Cycle and Reduce Patient Waiting Time | 180 (70.3) | **74** (66.7) | 106 (73.1) | **ns** | 125 (69.4) | 55 (72.4) | **ns** | 99 (65.6) | 81 (77.1) | **ns** | 128 (68.1) | 52 (76.5) | **ns** | 103 (69.6) | 77 (71.3) | ns | 127 (70.6) | 53 (69.7) | **ns** |
| Help Patients Understand Their Conditions More Scientifically | 143 (55.9) | **59** (53.2) | 84 (57.9) | **ns** | 102 (56.7) | 41 (53.9) | **ns** | 76 (50.3) | 67 (63.8) | **0.041** | 103 (54.8) | 40 (58.8) | **ns** | 88 (59.5) | 55 (50.9) | ns | 96 (53.3) | 47 (61.8) | **ns** |
| **What Position Do You Think Renal Pathology Should Occupy in the Standardized Training of Nephrologists (Resident/Specialist Training)?** | | | | | | | | | | | | | | | | | | | |
| Core Compulsory Content, Requiring Systematic and In-depth Learning | 81 (31.6) | **30** (27.0) | 51 (35.2) | **ns** | 51 (28.3) | 30 (39.5) | **0.105** | 44 (29.1) | 37 (35.2) | **ns** | 55 (29.3) | 26 (38.2) | **ns** | 49 (33.1) | 32 (29.6) | ns | 57 (31.7) | 24 (31.6) | **ns** |
| Important Content, Requiring Mastery of Basic Knowledge and Report Interpretation | 139 (54.3) | **63** (56.8) | 76 (52.4) | **ns** | 101 (56.1) | 38 (50.0) | **ns** | 84 (55.6) | 55 (52.4) | **ns** | 107 (56.9) | 32 (47.1) | **ns** | 82 (55.4) | 57 (52.8) | ns | 95 (52.8) | 44 (57.9) | **ns** |
| General Content, Understanding Suffices | 27 (10.5) | **14** (12.6) | 13 (9.0) | **ns** | 20 (11.1) | 7 (9.2) | **ns** | 19 (12.6) | 8 (7.6) | **ns** | 19 (10.1) | 8 (11.8) | **ns** | 14 (9.5) | 13 (12.0) | ns | 21 (11.7) | 6 (7.9) | **ns** |
| Non-key Content / No Need for Specialized Learning | 9 (3.5) | **4** (3.6) | 5 (3.4) | **ns** | 8 (4.4) | 1 (1.3) | **ns** | 4 (2.6) | 5 (4.8) | **ns** | 7 (3.7) | 2 (2.9) | **ns** | 4 (2.7) | 5 (4.6) | ns | 7 (3.9) | 2 (2.6) | **ns** |
| **What Do You Think Is the Appropriate Duration of Rotation?** | | | | | | | | | | | | | | | | | | | |
| ≥ 3 Months | 140 (54.7) | **59** (53.2) | 81 (55.9) | **ns** | 101 (56.1) | 39 (51.3) | **ns** | 91 (60.3) | 49 (46.7) | **0.041** | 112 (59.6) | 28 (41.2) | **0.011** | 78 (52.7) | 62 (57.4) | ns | 100 (55.6) | 40 (52.6) | **ns** |
| **What Do You Think Are the Main Values of Clinical Rotation for Pathologists?** | | | | | | | | | | | | | | | | | | | |
| Understand Clinical Needs, Make Reports More Aligned with Diagnosis and Treatment Decisions | 213 (83.2) | **84** (75.7) | 129 (89.0) | **0.007** | 149 (82.8) | 64 (84.2) | **ns** | 123 (81.5) | 90 (85.7) | **ns** | 157 (83.5) | 56 (82.4) | **ns** | 126 (85.1) | 87 (80.6) | ns | 149 (82.8) | 64 (84.2) | **ns** |
| Familiarize with Correlation Between Clinical Symptoms and Pathological Changes, Reduce Misdiagnosis | 211 (82.4) | **88** (79.3) | 123 (84.8) | **ns** | 146 (81.1) | 65 (85.5) | **ns** | 117 (77.5) | 94 (89.5) | **0.013** | 149 (79.3) | 62 (91.2) | **0.026** | 122 (82.4) | 89 (82.4) | ns | 146 (81.1) | 65 (85.5) | **ns** |
| Promote Communication Rapport with Clinicians | 152 (59.4) | **69** (62.2) | 83 (57.2) | **ns** | 99 (55.0) | 53 (69.7) | **0.036** | 77 (51.0) | 75 (71.4) | **0.001** | 105 (55.9) | 47 (69.1) | **0.062** | 84 (56.8) | 68 (63.0) | ns | 109 (60.6) | 43 (56.6) | **ns** |
| **What Is the Optimal Communication Frequency Between Clinicians and Pathologists?** | | | | | | | | | | | | | | | | | | | |
| ≥ 1 Time/Month | 229 (89.5) | **98** (88.3) | 131 (90.3) | **ns** | 161 (89.4) | 68 (89.5) | **ns** | 136 (90.1) | 93 (88.6) | **ns** | 168 (89.4) | 61 (89.7) | **ns** | 132 (89.2) | 97 (89.8) | ns | 159 (88.3) | 70 (92.1) | **ns** |
| **What Do You Think Is the Most Effective Clinical-Pathological Communication Method?** | | | | | | | | | | | | | | | | | | | |
| Joint Slide Review for Difficult Cases | 212 (82.8) | **84** (75.7) | 128 (88.3) | **0.012** | 150 (83.3) | 62 (81.6) | **ns** | 123 (81.5) | 89 (84.8) | **ns** | 155 (82.4) | 57 (83.8) | **ns** | 127 (85.8) | 85 (78.7) | ns | 148 (82.2) | 64 (84.2) | **ns** |
| Pathologists Participating in Clinical Ward Rounds | 172 (67.2) | **74** (66.7) | 98 (67.6) | **ns** | 119 (66.1) | 53 (69.7) | **ns** | 93 (61.6) | 79 (75.2) | **0.03** | 121 (64.4) | 51 (75.0) | **ns** | 98 (66.2) | 74 (68.5) | ns | 117 (65.0) | 55 (72.4) | **ns** |
| Online Communication Platform | 152 (59.4) | **68** (61.3) | 84 (57.9) | **ns** | 106 (58.9) | 46 (60.5) | **ns** | 84 (55.6) | 68 (64.8) | **ns** | 108 (57.4) | 44 (64.7) | **ns** | 86 (58.1) | 66 (61.1) | ns | 106 (58.9) | 46 (60.5) | **ns** |
| Regular Clinical-Pathological Joint Training | 129 (50.4) | **48** (43.2) | 81 (55.9) | 0.058 | 86 (47.8) | 43 (56.6) | **ns** | 68 (45.0) | 61 (58.1) | **0.043** | 94 (50.0) | 35 (51.5) | **ns** | 79 (53.4) | 50 (46.3) | ns | 89 (49.4) | 40 (52.6) | **ns** |
| ns, not significant; | | | | | | | | | | | | | | | | | | | |
