# Supplementary material for: Continuing medical education in renal pathology: current practices and needs among nephrologists
Source: BMC Med Educ. 2026 Feb 12;26:441. doi: 10.1186/s12909-026-08798-4 (PMC12997942; doi:10.1186/s12909-026-08798-4)
Supplement: Supplementary file 6 — Supplementary Material 6. [file 12909_2026_8798_MOESM6_ESM.docx]

**Supplemental Table 5. Univariate Logistic Regression Analysis of All Questionnaire Survey Results**

| **Observation indicators** | **Sex (male)** | | | **Age (≤40y)** | | | **Working time (≤10y)** | | | **Medical Professional Title (Attending Physician and Below)** | | | **Affiliation (Grade A Tertiary Hospital)** | | |
| --- | --- | --- | --- | --- | --- | --- | --- | --- | --- | --- | --- | --- | --- | --- | --- |
|  | **OR** | **95% CI** | **p** | **OR** | **95% CI** | **p** | **OR** | **95% CI** | **p** | **OR** | **95% CI** | **p** | **OR** | **95% CI** | **p** |
| Analysis of Importance and Existing Problems |  |  |  |  |  |  |  |  |  |  |  |  |  |  |  |
| Importance of Mastering Basic Knowledge of Renal Pathology (Extremely Important) | NS | NS | NS | NS | NS | NS | 2.05 | 1.11-3.80 | 0.022 | NS | NS | NS | NS | NS | NS |
| Ability to Independently Interpret Pathology Reports (Extremely Important) | 2.32 | 1.34-4.01 | 0.003 | NS | NS | NS | 1.93 | 1.09-3.41 | 0.024 | NS | NS | NS | NS | NS | NS |
| Analysis of Importance and Existing Problems |  | | | |  |  |  |  |  |  |  |  |  |  |  |
| Clarifying Disease Diagnosis (Extremely Important) | 2.57 | 1.41-4.72 | 0.002 | NS | NS | NS | 2.31 | 1.20-4.44 | 0.012 | NS | NS | NS | NS | NS | NS |
| Assessing Disease Activity and Severity (Extremely Important) | 1.84 | 1.05-3.24 | 0.034 | NS | NS | NS | NS | NS | NS | NS | NS | NS | 0.55 | 0.31-0.97 | 0.04 |
| Predicting Disease Progression and Prognosis (Extremely Important) | 2.25 | 1.29-3.92 | 0.004 | NS | NS | NS | 1.98 | 1.10-3.56 | 0.022 | NS | NS | NS | 0.49 | 0.28-0.85 | 0.011 |
| Guiding Individualized Treatment Regimen Selection (Extremely Important) | 2.25 | 1.28-3.95 | 0.005 | NS | NS | NS | 1.86 | 1.03-3.36 | 0.038 | NS | NS | NS | 0.53 | 0.30-0.92 | 0.025 |
| Evaluating Treatment Response (Extremely Important) | 2.32 | 1.34-4.01 | 0.003 | NS | NS | NS | 1.93 | 1.09-3.41 | 0.024 | NS | NS | NS | NS | NS | NS |
| Determining Eligibility for Clinical Trials (Extremely Important) | NS | NS | NS | NS | NS | NS | NS | NS | NS | NS | NS | NS | NS | NS | NS |
| **Importance of Renal Pathology in Various Diseases** |  |  |  |  |  |  |  |  |  |  |  |  |  |  |  |
| Primary Glomerular Diseases (e.g., IgA Nephropathy) (Extremely Important) | NS | NS | NS | NS | NS | NS | 2.3 | 1.27-4.15 | 0.006 | 1.98 | 1.01-3.91 | 0.048 | NS | NS | NS |
| Secondary Glomerular Diseases (e.g., Diabetic Nephropathy) (Extremely Important) | NS | NS | NS | 0.49 | 0.27-0.87 | 0.016 | 1.87 | 1.11-3.16 | 0.02 | NS | NS | NS | NS | NS | NS |
| Acute Kidney Injury (AKI) (When Etiological Differentiation is Required) (Extremely Important) | NS | NS | NS | NS | NS | NS | NS | NS | NS | NS | NS | NS | NS | NS | NS |
| Chronic Kidney Disease (CKD) (When Assessing Progression Risk) (Extremely Important) | NS | NS | NS | NS | NS | NS | NS | NS | NS | NS | NS | NS | NS | NS | NS |
| Post-Kidney Transplant Complications (e.g., Rejection) (Extremely Important) | 1.86 | 1.11-3.14 | 0.019 | NS | NS | NS | NS | NS | NS | NS | NS | NS | NS | NS | NS |
| Depth of Renal Pathology Knowledge Required for Non-Pathologist Clinicians |  | | | |  |  |  |  |  |  |  |  |  |  |  |
| Basic Pathological Terminology (e.g., "Mesangial Proliferation", "Crescent Formation") | 2.11 | 1.12-3.99 | 0.022 | NS | NS | NS | NS | NS | NS | NS | NS | NS | NS | NS | NS |
| Pathological Features of Common Diseases (e.g., IgA Nephropathy, Diabetic Nephropathy) | NS | NS | NS | NS | NS | NS | 5.8 | 1.69-19.92 | 0.005 | NS | NS | NS | NS | NS | NS |
| Differentiation of Complex Pathological Types (e.g., Lupus Nephritis Classification, Membranous Nephropathy Staging) | NS | NS | NS | NS | NS | NS | 2.79 | 1.56-4.97 | 0.001 | NS | NS | NS | NS | NS | NS |
| Clinical Significance of Immunofluorescence/Electron Microscopy Results | 2.58 | 1.53-4.34 | <0.001 | NS | NS | NS | 2.76 | 1.59-4.80 | <0.001 | NS | NS | NS | 0.54 | 0.32-0.91 | 0.02 |
| Main Difficulties in Interpreting Renal Pathology Reports |  | | | |  |  |  |  |  |  |  |  |  |  |  |
| Opaque and Hard-to-Understand Pathological Terminology | NS | NS | NS | NS | NS | NS | NS | NS | NS | NS | NS | NS | NS | NS | NS |
| Difficulty in Correlating Pathological Descriptions with Clinical Symptoms | NS | NS | NS | NS | NS | NS | NS | NS | NS | NS | NS | NS | 1.94 | 1.10-3.44 | 0.023 |
| Unclear Clinical Significance of Different Pathological Changes | NS | NS | NS | NS | NS | NS | NS | NS | NS | NS | NS | NS | NS | NS | NS |
| Lack of Effective Communication with Pathologists | NS | NS | NS | NS | NS | NS | 2.22 | 1.33-3.71 | 0.002 | 1.96 | 1.10-3.49 | 0.023 | NS | NS | NS |
| Current Status, Problems and Difficulties of Renal Pathology-related Training |  |  |  |  |  |  |  |  |  |  |  |  |  |  |  |
| Through which channels do you mainly learn and update renal pathology knowledge? |  | | |  |  |  |  |  |  |  |  |  |  |  |  |
| Medical School Courses | NS | NS | NS | 1.87 | 1.09-3.22 | 0.023 | NS | NS | NS | 0.54 | 0.31-0.94 | 0.031 | NS | NS | NS |
| Resident/Specialist Standardized Training | NS | NS | NS | 3.18 | 1.82-5.56 | <0.001 | 0.41 | 0.25-0.68 | 0.001 | 0.33 | 0.18-0.59 | <0.001 | 1.68 | 1.10-2.79 | 0.046 |
| Attending Academic Conferences/Lectures/Training Programs | NS | NS | NS | NS | NS | NS | 3.61 | 1.81-7.21 | <0.001 | 2.44 | 1.13-5.28 | 0.023 | NS | NS | NS |
| Reading Professional Books and Journal Literatures | NS | NS | NS | NS | NS | NS | 2.49 | 1.45-4.29 | 0.001 | NS | NS | NS | NS | NS | NS |
| Online Learning Resources (e.g., Online Courses, Databases, Pathology Atlases) | NS | NS | NS | NS | NS | NS | 1.74 | 1.03-2.93 | 0.038 | NS | NS | NS | NS | NS | NS |
| Communication/Joint Slide Review with Pathologists | NS | NS | NS | 0.58 | 0.34-0.99 | 0.048 | 1.87 | 1.12-3.11 | 0.016 | 1.83 | 1.04-3.20 | 0.035 | NS | NS | NS |
| Case Discussions (Departmental/MDT) | NS | NS | NS | NS | NS | NS | 1.88 | 1.13-3.13 | 0.014 | NS | NS | NS | NS | NS | NS |
| Self-study | NS | NS | NS | NS | NS | NS | NS | NS | NS | NS | NS | NS | NS | NS | NS |
| Do you currently participate in renal pathology-related training (e.g., academic conferences, pathology slide seminars)? |  | | |  |  |  |  |  |  |  |  |  |  |  |  |
| ≥ 1 Time/Year | NS | NS | NS | NS | NS | NS | 0.49 | 0.30-0.82 | 0.006 | NS | NS | NS | NS | NS | NS |
| Do you think the following abilities have improved after attending renal pathology-related training? |  | | |  |  |  |  |  |  |  |  |  |  |  |  |
| Accuracy of Pathology Report Interpretation | NS | NS | NS | NS | NS | NS | NS | NS | NS | NS | NS | NS | NS | NS | NS |
| Clinical-Pathological Correlation Analysis Ability | NS | NS | NS | NS | NS | NS | 2.61 | 1.29-5.27 | 0.008 | 2.58 | 1.10-6.06 | 0.029 | NS | NS | NS |
| Confidence in Diagnosis and Treatment of Complex Cases | NS | NS | NS | NS | NS | NS | 1.82 | 1.07-3.08 | 0.025 | 1.91 | 1.05-3.49 | 0.036 | NS | NS | NS |
| Communication Efficiency with Pathologists | NS | NS | NS | NS | NS | NS | 1.69 | 1.02-2.80 | 0.04 | NS | NS | NS | NS | NS | NS |
| No Significant Improvement Perceived | NS | NS | NS | NS | NS | NS | NS | NS | NS | NS | NS | NS | NS | NS | NS |
| What are the main difficulties you face in participating in renal pathology continuing medical education? |  | | |  |  |  |  |  |  |  |  |  |  |  |  |
| Time Conflict (Busy Clinical Work, Difficulty in Sparing Time) | NS | NS | NS | NS | NS | NS | NS | NS | NS | NS | NS | NS | NS | NS | NS |
| Training Resources Concentrated in Large Cities, Inconvenient for Grassroots Participation | NS | NS | NS | NS | NS | NS | NS | NS | NS | NS | NS | NS | 2.01 | 1.14-3.53 | 0.015 |
| Mismatch Between Training Content and Personal Needs (e.g., Tertiary Hospital Physicians Find Content Too Basic; Grassroots Physicians Find It Too Complex) | NS | NS | NS | NS | NS | NS | NS | NS | NS | NS | NS | NS | NS | NS | NS |
| Lack of Funding Support (e.g., Training Fees, Travel Expenses) | NS | NS | NS | NS | NS | NS | 2.18 | 1.31-3.62 | 0.003 | 1.96 | 1.11-3.45 | 0.02 | 2.56 | 1.54-4.27 | <0.001 |
| Single Training Format (e.g., Online Only, Lack of Practical Operation) | NS | NS | NS | NS | NS | NS | 2.34 | 1.38-3.96 | 0.002 | NS | NS | NS | NS | NS | NS |
| What do you think are the main problems in current renal pathology continuing medical education? |  | | | |  |  |  |  |  |  |  |  |  |  |  |
| Low Training Frequency (e.g., Only 1–2 Times/Year) | NS | NS | NS | NS | NS | NS | NS | NS | NS | NS | NS | NS | NS | NS | NS |
| Disconnection Between Content and Clinical Practice (e.g., Pure Theory, Lack of Case Analysis) | NS | NS | NS | NS | NS | NS | NS | NS | NS | NS | NS | NS | NS | NS | NS |
| Single Format (e.g., Offline Lectures Only, No Online Playback) | NS | NS | NS | NS | NS | NS | 1.74 | 1.02-2.97 | 0.041 | NS | NS | NS | NS | NS | NS |
| Lack of Targeting (e.g., Failing to Distinguish Needs of Physicians with Different Titles) | NS | NS | NS | NS | NS | NS | 2.52 | 1.50-4.25 | 0.001 | 1.84 | 1.03-3.27 | 0.039 | NS | NS | NS |
| Absence of Assessment Mechanism (No Feedback on Training Effects After Completion) | NS | NS | NS | NS | NS | NS | NS | NS | NS | NS | NS | NS | NS | NS | NS |
| **Importance, Methods and Approaches of Continuing Medical Education** |  |  |  |  |  |  |  |  |  |  |  |  |  |  |  |
| **What Renal Pathology Training Content Do You Think Needs the Most Strengthening?** |  | | |  |  |  |  |  |  |  |  |  |  |  |  |
| Pathological Features of Common Renal Diseases | NS | NS | NS | NS | NS | NS | NS | NS | NS | NS | NS | NS | NS | NS | NS |
| Systematic Interpretation Methods of Pathology Reports | NS | NS | NS | NS | NS | NS | 2.12 | 1.01-4.45 | 0.047 | NS | NS | NS | NS | NS | NS |
| Clinical-Pathological Case Analysis | NS | NS | NS | NS | NS | NS | 2.24 | 1.16-4.30 | 0.016 | NS | NS | NS | NS | NS | NS |
| Application of New Renal Pathology Technologies | NS | NS | NS | 0.53 | 0.30-0.96 | 0.035 | 2.25 | 1.32-3.86 | 0.003 | 1.97 | 1.07-3.63 | 0.03 | NS | NS | NS |
| What Do You Think Is the Most Effective Training Format? |  | | |  |  |  |  |  |  |  |  |  |  |  |  |
| Clinical-Pathological Case Conferences | NS | NS | NS | NS | NS | NS | NS | NS | NS | NS | NS | NS | NS | NS | NS |
| Practical Pathology Slide Review | NS | NS | NS | NS | NS | NS | NS | NS | NS | NS | NS | NS | NS | NS | NS |
| Online Recorded Courses | NS | NS | NS | NS | NS | NS | NS | NS | NS | NS | NS | NS | NS | NS | NS |
| Face-to-face Q&A with Experts | 1.71 | 1.03-2.82 | 0.038 | NS | NS | NS | 1.75 | 1.04-2.92 | 0.033 | NS | NS | NS | NS | NS | NS |
| Skill Assessment | NS | NS | NS | NS | NS | NS | 1.74 | 1.03-2.95 | 0.038 | NS | NS | NS | NS | NS | NS |
| What Training Cycle Do You Expect for Renal Pathology-related Programs? |  | | |  |  |  |  |  |  |  |  |  |  |  |  |
| Once a Month | NS | NS | NS | NS | NS | NS | NS | NS | NS | NS | NS | NS | NS | NS | NS |
| Once a Quarter | NS | NS | NS | NS | NS | NS | NS | NS | NS | NS | NS | NS | NS | NS | NS |
| Once Every 6 Months / 1-2 Times a Year | NS | NS | NS | 0.44 | 0.20-0.98 | 0.044 | NS | NS | NS | NS | NS | NS | NS | NS | NS |
| On-demand Implementation | NS | NS | NS | NS | NS | NS | NS | NS | NS | NS | NS | NS | NS | NS | NS |
| In Which Aspects Do You Most Want to Obtain More Renal Pathology Training or Resources? |  | | | |  |  |  |  |  |  |  |  |  |  |  |
| Key Points and Pitfalls of Pathological Diagnosis for Common and Rare Renal Diseases | 2.46 | 1.30-4.65 | 0.006 | NS | NS | NS | 2.22 | 1.11-4.42 | 0.024 | NS | NS | NS | NS | NS | NS |
| In-depth Interpretation and Clinical Significance of Renal Biopsy Reports | NS | NS | NS | NS | NS | NS | NS | NS | NS | NS | NS | NS | NS | NS | NS |
| Detailed Explanation and Application of Pathological Classification/Scoring Systems | NS | NS | NS | NS | NS | NS | 1.86 | 1.08-3.21 | 0.024 | NS | NS | NS | NS | NS | NS |
| Interpretation Skills of Light Microscopy, Immunofluorescence and Electron Microscopy Images | NS | NS | NS | NS | NS | NS | NS | NS | NS | NS | NS | NS | NS | NS | NS |
| Integration of Latest Clinical Guidelines and Renal Pathology Knowledge | NS | NS | NS | NS | NS | NS | NS | NS | NS | NS | NS | NS | 0.56 | 0.33-0.93 | 0.025 |
| How to Better Communicate and Collaborate with Pathologists | NS | NS | NS | NS | NS | NS | NS | NS | NS | 3.35 | 1.15-9.73 | 0.026 | NS | NS | NS |
| Latest Research Progress in Renal Pathology | 1.84 | 1.11-3.04 | 0.017 | NS | NS | NS | 2.41 | 1.44-4.01 | 0.001 | NS | NS | NS | 0.59 | 0.36-0.98 | 0.042 |
| Recommendations for Online Pathology Atlases/Database Resources | NS | NS | NS | NS | NS | NS | 2.14 | 1.28-3.59 | 0.004 | NS | NS | NS | NS | NS | NS |
| Practical Slide Review Workshops | NS | NS | NS | NS | NS | NS | 2.24 | 1.32-3.81 | 0.003 | NS | NS | NS | NS | NS | NS |
| **What Impacts Do You Think Systematic Renal Pathology Continuing Education May Have on Patient Outcomes?** |  | | | |  |  |  |  |  |  |  |  |  |  |  |
| Reduce Misdiagnosis Rate and Unnecessary Treatment | 2.27 | 1.17-4.37 | 0.015 | 0.38 | 0.16-0.89 | 0.026 | NS | NS | NS | NS | NS | NS | NS | NS | NS |
| Improve Matching Degree Between Treatment Plans and Pathological Types, Enhance Therapeutic Effect | NS | NS | NS | NS | NS | NS | 2.66 | 1.25-5.67 | 0.011 | 4.19 | 1.44-12.21 | 0.009 | NS | NS | NS |
| Shorten Diagnosis Cycle and Reduce Patient Waiting Time | NS | NS | NS | NS | NS | NS | 1.77 | 1.01-3.12 | 0.047 | NS | NS | NS | NS | NS | NS |
| Help Patients Understand Their Conditions More Scientifically | NS | NS | NS | NS | NS | NS | 1.74 | 1.04-2.90 | 0.033 | NS | NS | NS | NS | NS | NS |
| What Position Do You Think Renal Pathology Should Occupy in the Standardized Training of Nephrologists (Resident/Specialist Training)? |  | | | |  |  |  |  |  |  |  |  |  |  |  |
| Core Compulsory Content, Requiring Systematic and In-depth Learning | NS | NS | NS | NS | NS | NS | NS | NS | NS | NS | NS | NS | NS | NS | NS |
| Important Content, Requiring Mastery of Basic Knowledge and Report Interpretation | NS | NS | NS | NS | NS | NS | NS | NS | NS | NS | NS | NS | NS | NS | NS |
| General Content, Understanding Suffices | NS | NS | NS | NS | NS | NS | NS | NS | NS | NS | NS | NS | NS | NS | NS |
| Non-key Content / No Need for Specialized Learning | NS | NS | NS | NS | NS | NS | NS | NS | NS | NS | NS | NS | NS | NS | NS |
| What Do You Think Is the Appropriate Duration of Rotation? |  | | | |  |  |  |  |  |  |  |  |  |  |  |
| ≥ 3 Months | NS | NS | NS | NS | NS | NS | 1.73 | 1.05-2.87 | 0.032 | 2.11 | 1.20-3.70 | 0.01 | NS | NS | NS |
| What Do You Think Are the Main Values of Clinical Rotation for Pathologists? |  | | | |  |  |  |  |  |  |  |  |  |  |  |
| Understand Clinical Needs, Make Reports More Aligned with Diagnosis and Treatment Decisions | 2.59 | 1.32-5.10 | 0.006 | NS | NS | NS | NS | NS | NS | NS | NS | NS | NS | NS | NS |
| Familiarize with Correlation Between Clinical Symptoms and Pathological Changes, Reduce Misdiagnosis | NS | NS | NS | NS | NS | NS | 2.48 | 1.19-5.16 | 0.015 | 2.71 | 1.09-6.71 | 0.032 | NS | NS | NS |
| Promote Communication Rapport with Clinicians | NS | NS | NS | 0.53 | 0.30-0.94 | 0.029 | 2.88 | 1.63-4.77 | <0.001 | NS | NS | NS | NS | NS | NS |
| What Is the Optimal Communication Frequency Between Clinicians and Pathologists? |  | | | |  |  |  |  |  |  |  |  |  |  |  |
| ≥ 1 Time/Month | NS | NS | NS | NS | NS | NS | NS | NS | NS | NS | NS | NS | NS | NS | NS |
| What Do You Think Is the Most Effective Clinical-Pathological Communication Method? |  | | | |  |  |  |  |  |  |  |  |  |  |  |
| Joint Slide Review for Difficult Cases | 2.42 | 1.24-4.71 | 0.009 | NS | NS | NS | NS | NS | NS | NS | NS | NS | NS | NS | NS |
| Pathologists Participating in Clinical Ward Rounds | NS | NS | NS | NS | NS | NS | 1.89 | 1.09-3.29 | 0.023 | NS | NS | NS | NS | NS | NS |
| Online Communication Platform | NS | NS | NS | NS | NS | NS | NS | NS | NS | NS | NS | NS | NS | NS | NS |
| Regular Clinical-Pathological Joint Training | 1.66 | 1.01-2.73 | 0.046 | NS | NS | NS | 1.69 | 1.02-2.80 | 0.04 | NS | NS | NS | NS | NS | NS |
| **Basic Situation of Primary Medical Institutions, Third-Party Institutions and AI** |  |  |  |  |  |  |  |  |  |  |  |  |  |  |  |
| What do you think are the core needs of primary medical institutions for renal pathology? |  | | | |  |  |  |  |  |  |  |  |  |  |  |
| Channels for rapid specimen transportation to external institutions | NS | NS | NS | NS | NS | NS | NS | NS | NS | NS | NS | NS | NS | NS | NS |
| Simplified pathology report | NS | NS | NS | NS | NS | NS | NS | NS | NS | NS | NS | NS | NS | NS | NS |
| Telepathology consultation support | NS | NS | NS | NS | NS | NS | 2.1 | 1.16-3.81 | 0.014 | NS | NS | NS | NS | NS | NS |
| Renal pathology knowledge training tailored for primary institutions | 2.23 | 1.30-3.82 | 0.004 | NS | NS | NS | 2.1 | 1.19-3.71 | 0.01 | 2.04 | 1.05-3.94 | 0.035 | NS | NS | NS |
| What are the main restrictive factors for primary institutions to carry out renal pathology-related work? |  | | | |  |  |  |  |  |  |  |  |  |  |  |
| Lack of specimen processing equipment | NS | NS | NS | NS | NS | NS | NS | NS | NS | 0.49 | 0.27-0.92 | 0.025 | 1.86 | 1.01-3.42 | 0.047 |
| Insufficient technical personnel | NS | NS | NS | NS | NS | NS | 2.34 | 1.15-4.76 | 0.019 | NS | NS | NS | NS | NS | NS |
| High cost of external specimen transportation | NS | NS | NS | NS | NS | NS | NS | NS | NS | NS | NS | NS | NS | NS | NS |
| Unsmooth cooperation mechanism with pathology departments of superior hospitals | NS | NS | NS | NS | NS | NS | 2.06 | 1.23-3.46 | 0.006 | NS | NS | NS | NS | NS | NS |
| Does your institution rely on third-party pathological testing institutions? |  | | | |  |  |  |  |  |  |  |  |  |  |  |
| Yes (regularly send to professional pathological testing companies) | 0.47 | 0.28-0.77 | 0.003 | 0.49 | 0.29-0.85 | 0.011 | 2.95 | 1.76-4.95 | <0.001 | 2.58 | 1.46-4.55 | 0.001 | NS | NS | NS |
| Yes (regularly send to superior hospitals, e.g., primary hospitals send to tertiary hospitals) | NS | NS | NS | NS | NS | NS | NS | NS | NS | NS | NS | NS | 1.97 | 1.13-3.42 | 0.016 |
| No (with its own pathology department) | 3.39 | 1.72-6.68 | <0.001 | 2.09 | 1.02-4.29 | 0.045 | 0.47 | 0.25-0.88 | 0.02 | NS | NS | NS | 0.35 | 0.18-0.68 | 0.002 |
| What does your trust in third-party institutions mainly depend on? |  | | |  |  |  |  |  |  |  |  |  |  |  |  |
| Testing qualifications (e.g., certified laboratories) | NS | NS | NS | NS | NS | NS | NS | NS | NS | NS | NS | NS | NS | NS | NS |
| Timeliness of reports | NS | NS | NS | NS | NS | NS | 2.02 | 1.18-3.47 | 0.011 | NS | NS | NS | NS | NS | NS |
| Ability to provide clinical interpretation suggestions | NS | NS | NS | NS | NS | NS | 2.99 | 1.72-5.23 | <0.001 | 2.36 | 1.25-4.43 | 0.008 | NS | NS | NS |
| Reasonableness of prices | NS | NS | NS | NS | NS | NS | 1.81 | 1.09-3.01 | 0.022 | NS | NS | NS | NS | NS | NS |
| What do you think is the potential value of AI in renal pathology? |  | | |  |  |  |  |  |  |  |  |  |  |  |  |
| Rapid preliminary screening (e.g., identifying typical lesions, reducing manual workload) | NS | NS | NS | NS | NS | NS | NS | NS | NS | NS | NS | NS | NS | NS | NS |
| Quantitative analysis (e.g., automatic counting of glomerulosclerosis ratio) | NS | NS | NS | NS | NS | NS | NS | NS | NS | NS | NS | NS | NS | NS | NS |
| Assisting primary physicians in report interpretation (e.g., AI generates popularized conclusions) | NS | NS | NS | NS | NS | NS | 1.93 | 1.09-3.41 | 0.024 | NS | NS | NS | NS | NS | NS |
| No significant value (relying on pathologists' experience is more reliable) | NS | NS | NS | NS | NS | NS | NS | NS | NS | NS | NS | NS | 3.51 | 1.53-8.06 | 0.003 |
| What are your main concerns about the application of AI? |  | | |  |  |  |  |  |  |  |  |  |  |  |  |
| Misdiagnosis risk (especially for rare/complex cases) | NS | NS | NS | NS | NS | NS | NS | NS | NS | NS | NS | NS | NS | NS | NS |
| Data privacy leakage (patients' pathological images) | NS | NS | NS | NS | NS | NS | NS | NS | NS | NS | NS | NS | NS | NS | NS |
| Over-reliance on AI, weakening physicians' subjective judgment ability | 1.91 | 1.11-3.31 | 0.021 | NS | NS | NS | 3.74 | 1.98-7.08 | <0.001 | NS | NS | NS | NS | NS | NS |
| Lack of unified standards, inconsistent results among different AI systems | NS | NS | NS | NS | NS | NS | 1.76 | 1.05-2.95 | 0.033 | NS | NS | NS | NS | NS | NS |
| **ns, not significant;** | | | | | | | | | | | | | | | |
