# Supplementary material for: Continuing medical education in renal pathology: current practices and needs among nephrologists
Source: BMC Med Educ. 2026 Feb 12;26:441. doi: 10.1186/s12909-026-08798-4 (PMC12997942; doi:10.1186/s12909-026-08798-4)
Supplement: Supplementary file 7 — Supplementary Material 7. [file 12909_2026_8798_MOESM7_ESM.docx]

**Supplemental Table 6. Multivariate Logistic Regression Analysis of All Questionnaire Survey Results**

| **Observation indicators** | **Sex (male)**❋ | | | **Age (≤40y)**※ | | | **Working time (≤10y)** † | | | **Medical Professional Title (Attending Physician and Below)**ƫ | | | **Affiliation (Grade A Tertiary Hospital)**§ | | |
| --- | --- | --- | --- | --- | --- | --- | --- | --- | --- | --- | --- | --- | --- | --- | --- |
|  | **OR** | **95% CI** | **p** | **OR** | **95% CI** | **p** | **OR** | **95% CI** | **p** | **OR** | **95% CI** | **p** | **OR** | **95% CI** | **p** |
| **Analysis of Importance and Existing Problems** | | | | | | | | | | | | | | | |
| Importance of Mastering Basic Knowledge of Renal Pathology (Extremely Important) | NS | NS | NS | NS | NS | NS | 2.5 | 1.08-5.77 | 0.032 | NS | NS | NS | NS | NS | NS |
| Ability to Independently Interpret Pathology Reports (Extremely Important) | 2.62 | 1.48-4.64 | 0.001 | NS | NS | NS | 2.68 | 1.19-6.03 | 0.017 | NS | NS | NS | NS | NS | NS |
| **Analysis of Importance and Existing Problems** |  | | | |  |  |  |  |  |  |  |  |  |  |  |
| Clarifying Disease Diagnosis (Extremely Important) | 2.91 | 1.54-5.48 | 0.001 | NS | NS | NS | 3.35 | 1.34-8.38 | 0.01 | NS | NS | NS | NS | NS | NS |
| Assessing Disease Activity and Severity (Extremely Important) | 1.97 | 1.09-3.55 | 0.023 | NS | NS | NS | NS | NS | NS | NS | NS | NS | 0.55 | 0.31-0.98 | 0.043 |
| Predicting Disease Progression and Prognosis (Extremely Important) | 2.53 | 1.41-4.55 | 0.002 | NS | NS | NS | NS | NS | NS | NS | NS | NS | 0.47 | 0.26-0.85 | 0.012 |
| Guiding Individualized Treatment Regimen Selection (Extremely Important) | 2.46 | 1.37-4.43 | 0.003 | NS | NS | NS | NS | NS | NS | NS | NS | NS | 0.51 | 0.28-0.92 | 0.026 |
| Evaluating Treatment Response (Extremely Important) | 2.59 | 1.47-4.59 | 0.001 | NS | NS | NS | NS | NS | NS | NS | NS | NS | NS | NS | NS |
| Determining Eligibility for Clinical Trials (Extremely Important) | 1.73 | 1.02-2.92 | 0.040 | NS | NS | NS | NS | NS | NS | NS | NS | NS | NS | NS | NS |
| **Importance of Renal Pathology in Various Diseases** | | | | | | | | | | | | | | | |
| Primary Glomerular Diseases (e.g., IgA Nephropathy) (Extremely Important) | NS | NS | NS | NS | NS | NS | 2.26 | 1.03-4.95 | 0.041 | NS | NS | NS | NS | NS | NS |
| Secondary Glomerular Diseases (e.g., Diabetic Nephropathy) (Extremely Important) | NS | NS | NS | NS | NS | NS | NS | NS | NS | NS | NS | NS | NS | NS | NS |
| Acute Kidney Injury (AKI) (When Etiological Differentiation is Required) (Extremely Important) | NS | NS | NS | NS | NS | NS | NS | NS | NS | NS | NS | NS | NS | NS | NS |
| Chronic Kidney Disease (CKD) (When Assessing Progression Risk) (Extremely Important) | NS | NS | NS | NS | NS | NS | NS | NS | NS | NS | NS | NS | NS | NS | NS |
| Post-Kidney Transplant Complications (e.g., Rejection) (Extremely Important) | 1.95 | 1.94-3.32 | 0.014 | NS | NS | NS | NS | NS | NS | NS | NS | NS | NS | NS | NS |
| **Depth of Renal Pathology Knowledge Required for Non-Pathologist Clinicians** | | | | | | | | | | | | | | | |
| Basic Pathological Terminology (e.g., "Mesangial Proliferation", "Crescent Formation") | 2.26 | 1.18-4.34 | 0.014 | NS | NS | NS | NS | NS | NS | NS | NS | NS | NS | NS | NS |
| Pathological Features of Common Diseases (e.g., IgA Nephropathy, Diabetic Nephropathy) | 2.65 | 1.06-6.64 | 0.037 | 0.1 | 0.02-0.45 | 0.002 | 22.21 | 3.87-127.49 | 0.001 | NS | NS | NS | NS | NS | NS |
| Differentiation of Complex Pathological Types (e.g., Lupus Nephritis Classification, Membranous Nephropathy Staging) | NS | NS | NS | NS | NS | NS | 3.72 | 1.65-8.41 | 0.002 | NS | NS | NS | NS | NS | NS |
| Clinical Significance of Immunofluorescence/Electron Microscopy Results | 3.24 | 1.83-5.75 | <0.001 | NS | NS | NS | 4.67 | 2.06-10.65 | <0.001 | NS | NS | NS | 0.5 | 0.28-0.88 | 0.017 |
| **Main Difficulties in Interpreting Renal Pathology Reports** | | | | | | | | | | | | | | | |
| Opaque and Hard-to-Understand Pathological Terminology | NS | NS | NS | NS | NS | NS | NS | NS | NS | NS | NS | NS | NS | NS | NS |
| Difficulty in Correlating Pathological Descriptions with Clinical Symptoms | NS | NS | NS | NS | NS | NS | NS | NS | NS | NS | NS | NS | 2.12 | 1.17-3.82 | 0.013 |
| Unclear Clinical Significance of Different Pathological Changes | NS | NS | NS | 0.39 | 0.17-0.94 | 0.035 | NS | NS | NS | NS | NS | NS | NS | NS | NS |
| Lack of Effective Communication with Pathologists | NS | NS | NS | NS | NS | NS | 2.47 | 1.23-4.96 | 0.011 | NS | NS | NS | NS | NS | NS |
| **Current Status, Problems and Difficulties of Renal Pathology-related Training** | | | | | | | | | | | | | | | |
| **Through which channels do you mainly learn and update renal pathology knowledge?** | | | | | | | | | | | | | | | |
| Medical School Courses | NS | NS | NS | NS | NS | NS | NS | NS | NS | NS | NS | NS | NS | NS | NS |
| Resident/Specialist Standardized Training | NS | NS | NS | 0.45 | 0.21-0.99 | 0.046 | NS | NS | NS | NS | NS | NS | 1.98 | 1.15-3.43 | 0.014 |
| Attending Academic Conferences/Lectures/Training Programs | NS | NS | NS | 0.33 | 0.12-0.97 | 0.043 | 5.41 | 1.95-14.96 | 0.001 | NS | NS | NS | NS | NS | NS |
| Reading Professional Books and Journal Literatures | NS | NS | NS | 0.35 | 0.14-0.86 | 0.023 | 4.25 | 1.88-9.60 | <0.001 | NS | NS | NS | NS | NS | NS |
| Online Learning Resources (e.g., Online Courses, Databases, Pathology Atlases) | NS | NS | NS | 0.24 | 0.09-0.59 | 0.002 | 2.76 | 1.27-6.01 | 0.011 | NS | NS | NS | NS | NS | NS |
| Communication/Joint Slide Review with Pathologists | NS | NS | NS | NS | NS | NS | NS | NS | NS | NS | NS | NS | NS | NS | NS |
| Case Discussions (Departmental/MDT) | NS | NS | NS | NS | NS | NS | NS | NS | NS | NS | NS | NS | NS | NS | NS |
| Self-study | NS | NS | NS | NS | NS | NS | NS | NS | NS | NS | NS | NS | NS | NS | NS |
| **Do you currently participate in renal pathology-related training (e.g., academic conferences, pathology slide seminars)?** | | | | | | | | | | | | | | | |
| ≥ 1 Time/Year | 0.58 | 0.35-0.99 | 0.044 | NS | NS | NS | 0.4 | 0.20-0.79 | 0.009 | NS | NS | NS | NS | NS | NS |
| **Do you think the following abilities have improved after attending renal pathology-related training?** | | | | | | | | | | | | | | | |
| Accuracy of Pathology Report Interpretation | NS | NS | NS | NS | NS | NS | NS | NS | NS | NS | NS | NS | NS | NS | NS |
| Clinical-Pathological Correlation Analysis Ability | NS | NS | NS | 0.33 | 0.11-0.95 | 0.04 | 3.54 | 1.30-9.64 | 0.014 | NS | NS | NS | NS | NS | NS |
| Confidence in Diagnosis and Treatment of Complex Cases | NS | NS | NS | 0.33 | 0.14-0.79 | 0.012 | 2.29 | 1.08-4.83 | 0.03 | 2.47 | 1.09-5.56 | 0.029 | 1.73 | 1.01-2.98 | 0.048 |
| Communication Efficiency with Pathologists | NS | NS | NS | NS | NS | NS | NS | NS | NS | NS | NS | NS | NS | NS | NS |
| No Significant Improvement Perceived | NS | NS | NS | NS | NS | NS | NS | NS | NS | NS | NS | NS | 3.27 | 1.49-7.17 | 0.003 |
| **What are the main difficulties you face in participating in renal pathology continuing medical education?** | | | | | | | | | | | | | | | |
| Time Conflict (Busy Clinical Work, Difficulty in Sparing Time) | NS | NS | NS | NS | NS | NS | NS | NS | NS | NS | NS | NS | NS | NS | NS |
| Training Resources Concentrated in Large Cities, Inconvenient for Grassroots Participation | NS | NS | NS | 0.29 | 0.12-0.72 | 0.008 | NS | NS | NS | NS | NS | NS | 2.21 | 1.22-3.98 | 0.009 |
| Mismatch Between Training Content and Personal Needs (e.g., Tertiary Hospital Physicians Find Content Too Basic; Grassroots Physicians Find It Too Complex) | NS | NS | NS | NS | NS | NS | NS | NS | NS | NS | NS | NS | NS | NS | NS |
| Lack of Funding Support (e.g., Training Fees, Travel Expenses) | NS | NS | NS | 0.39 | 0.17-0.91 | 0.029 | 2.51 | 1.23-5.11 | 0.012 | NS | NS | NS | 2.74 | 1.60-4.69 | <0.001 |
| Single Training Format (e.g., Online Only, Lack of Practical Operation) | NS | NS | NS | NS | NS | NS | 3.57 | 1.76-7.25 | <0.001 | NS | NS | NS | NS | NS | NS |
| **What do you think are the main problems in current renal pathology continuing medical education?** | | | | | | | | | | | | | | | |
| Low Training Frequency (e.g., Only 1–2 Times/Year) | NS | NS | NS | NS | NS | NS | NS | NS | NS | NS | NS | NS | NS | NS | NS |
| Disconnection Between Content and Clinical Practice (e.g., Pure Theory, Lack of Case Analysis) | NS | NS | NS | 0.34 | 0.14-0.81 | 0.015 | NS | NS | NS | NS | NS | NS | NS | NS | NS |
| Single Format (e.g., Offline Lectures Only, No Online Playback) | NS | NS | NS | 0.32 | 0.13-0.77 | 0.011 | 3.52 | 1.56-7.96 | 0.003 | NS | NS | NS | NS | NS | NS |
| Lack of Targeting (e.g., Failing to Distinguish Needs of Physicians with Different Titles) | 1.82 | 1.07-3.09 | 0.027 | NS | NS | NS | 3.57 | 1.70-7.50 | 0.001 | NS | NS | NS | NS | NS | NS |
| Absence of Assessment Mechanism (No Feedback on Training Effects After Completion) | NS | NS | NS | NS | NS | NS | 2.13 | 1.04-4.34 | 0.038 | NS | NS | NS | NS | NS | NS |
| **Importance, Methods and Approaches of Continuing Medical Education** | | | | | | | | | | | | | | | |
| **What Renal Pathology Training Content Do You Think Needs the Most Strengthening?** | | | | | | | | | | | | | | | |
| Pathological Features of Common Renal Diseases | NS | NS | NS | NS | NS | NS | NS | NS | NS | NS | NS | NS | NS | NS | NS |
| Systematic Interpretation Methods of Pathology Reports | NS | NS | NS | NS | NS | NS | 3.45 | 1.18-10.08 | 0.023 | NS | NS | NS | NS | NS | NS |
| Clinical-Pathological Case Analysis | NS | NS | NS | 0.24 | 0.08-0.68 | 0.007 | 4.31 | 1.57-11.81 | 0.004 | NS | NS | NS | NS | NS | NS |
| Application of New Renal Pathology Technologies | NS | NS | NS | NS | NS | NS | 2.06 | 1.01-4.20 | 0.047 | NS | NS | NS | NS | NS | NS |
| **What Do You Think Is the Most Effective Training Format?** | | | | | | | | | | | | | | | |
| Clinical-Pathological Case Conferences | NS | NS | NS | NS | NS | NS | NS | NS | NS | NS | NS | NS | NS | NS | NS |
| Practical Pathology Slide Review | NS | NS | NS | NS | NS | NS | 2.62 | 1.06-6.45 | 0.037 | NS | NS | NS | NS | NS | NS |
| Online Recorded Courses | NS | NS | NS | 0.38 | 0.17-0.89 | 0.026 | 2.19 | 1.04-4.62 | 0.039 | NS | NS | NS | NS | NS | NS |
| Face-to-face Q&A with Experts | 1.88 | 1.11-3.17 | 0.019 | 0.42 | 0.18-0.96 | 0.4 | 3.56 | 1.64-7.71 | 0.001 | NS | NS | NS | NS | NS | NS |
| Skill Assessment | NS | NS | NS | NS | NS | NS | 2.45 | 1.22-4.91 | 0.012 | NS | NS | NS | NS | NS | NS |
| **What Training Cycle Do You Expect for Renal Pathology-related Programs?** | | | | | | | | | | | | | | | |
| Once a Month | NS | NS | NS | NS | NS | NS | NS | NS | NS | NS | NS | NS | NS | NS | NS |
| Once a Quarter | NS | NS | NS | NS | NS | NS | NS | NS | NS | NS | NS | NS | NS | NS | NS |
| Once Every 6 Months / 1-2 Times a Year | NS | NS | NS | NS | NS | NS | NS | NS | NS | NS | NS | NS | NS | NS | NS |
| On-demand Implementation | NS | NS | NS | NS | NS | NS | NS | NS | NS | NS | NS | NS | NS | NS | NS |
| **In Which Aspects Do You Most Want to Obtain More Renal Pathology Training or Resources?** | | | | | | | | | | | | | | | |
| Key Points and Pitfalls of Pathological Diagnosis for Common and Rare Renal Diseases | 2.79 | 1.43-5.42 | 0.003 | NS | NS | NS | NS | NS | NS | NS | NS | NS | NS | NS | NS |
| In-depth Interpretation and Clinical Significance of Renal Biopsy Reports | NS | NS | NS | 0.11 | 0.03-0.35 | <0.001 | 4.79 | 1.58-14.53 | 0.006 | NS | NS | NS | NS | NS | NS |
| Detailed Explanation and Application of Pathological Classification/Scoring Systems | NS | NS | NS | NS | NS | NS | NS | NS | NS | NS | NS | NS | NS | NS | NS |
| Interpretation Skills of Light Microscopy, Immunofluorescence and Electron Microscopy Images | NS | NS | NS | 0.35 | 0.15-0.86 | 0.022 | 2.65 | 1.18-5.91 | 0.018 | NS | NS | NS | NS | NS | NS |
| Integration of Latest Clinical Guidelines and Renal Pathology Knowledge | NS | NS | NS | 0.35 | 0.15-0.84 | 0.018 | 3.34 | 1.51-7.40 | 0.003 | NS | NS | NS | 0.56 | 0.33-0.96 | 0.034 |
| How to Better Communicate and Collaborate with Pathologists | NS | NS | NS | NS | NS | NS | 2.62 | 1.31-5.24 | 0.006 | NS | NS | NS | NS | NS | NS |
| Latest Research Progress in Renal Pathology | 2.14 | 1.25-3.66 | 0.006 | NS | NS | NS | 3.46 | 1.68-7.13 | 0.001 | NS | NS | NS | 0.55 | 0.32-0.94 | 0.029 |
| Recommendations for Online Pathology Atlases/Database Resources | NS | NS | NS | NS | NS | NS | 3.56 | 1.73-7.28 | 0.001 | NS | NS | NS | 0.55 | 0.32-0.96 | 0.034 |
| Practical Slide Review Workshops | NS | NS | NS | NS | NS | NS | 3.27 | 1.61-6.65 | 0.001 | NS | NS | NS | NS | NS | NS |
| **What Impacts Do You Think Systematic Renal Pathology Continuing Education May Have on Patient Outcomes?** | | | | | | | | | | | | | | | |
| Reduce Misdiagnosis Rate and Unnecessary Treatment | 2.64 | 1.33-5.21 | 0.005 | NS | NS | NS | NS | NS | NS | NS | NS | NS | NS | NS | NS |
| Improve Matching Degree Between Treatment Plans and Pathological Types, Enhance Therapeutic Effect | NS | NS | NS | 0.21 | 0.068-0.65 | 0.007 | 3.59 | 1.21-10.63 | 0.021 | 5.44 | 1.52-19.44 | 0.009 | NS | NS | NS |
| Shorten Diagnosis Cycle and Reduce Patient Waiting Time | NS | NS | NS | NS | NS | NS | NS | NS | NS | NS | NS | NS | NS | NS | NS |
| Help Patients Understand Their Conditions More Scientifically | NS | NS | NS | 0.43 | 0.19-0.98 | 0.044 | 3.05 | 1.45-6.39 | 0.003 | NS | NS | NS | NS | NS | NS |
| **What Position Do You Think Renal Pathology Should Occupy in the Standardized Training of Nephrologists (Resident/Specialist Training)?** | | | | | | | | | | | | | | | |
| Core Compulsory Content, Requiring Systematic and In-depth Learning | NS | NS | NS | NS | NS | NS | NS | NS | NS | NS | NS | NS | NS | NS | NS |
| Important Content, Requiring Mastery of Basic Knowledge and Report Interpretation | NS | NS | NS | NS | NS | NS | NS | NS | NS | NS | NS | NS | NS | NS | NS |
| General Content, Understanding Suffices | NS | NS | NS | NS | NS | NS | NS | NS | NS | NS | NS | NS | NS | NS | NS |
| Non-key Content / No Need for Specialized Learning | NS | NS | NS | NS | NS | NS | NS | NS | NS | NS | NS | NS | NS | NS | NS |
| **What Do You Think Is the Appropriate Duration of Rotation?** | | | | | | | | | | | | | | | |
| ≥ 3 Months | NS | NS | NS | NS | NS | NS | NS | NS | NS | 2.29 | 1.08-4.83 | 0.03 | NS | NS | NS |
| **What Do You Think Are the Main Values of Clinical Rotation for Pathologists?** | | | | | | | | | | | | | | | |
| Understand Clinical Needs, Make Reports More Aligned with Diagnosis and Treatment Decisions | 2.67 | 1.34-5.33 | 0.005 | NS | NS | NS | NS | NS | NS | NS | NS | NS | NS | NS | NS |
| Familiarize with Correlation Between Clinical Symptoms and Pathological Changes, Reduce Misdiagnosis | NS | NS | NS | NS | NS | NS | 2.99 | 1.09-8.17 | 0.033 | NS | NS | NS | NS | NS | NS |
| Promote Communication Rapport with Clinicians | NS | NS | NS | NS | NS | NS | 2.89 | 1.40-5.96 | 0.004 | NS | NS | NS | NS | NS | NS |
| **What Is the Optimal Communication Frequency Between Clinicians and Pathologists?** | | | | | | | | | | | | | | | |
| ≥ 1 Time/Month | NS | NS | NS | NS | NS | NS | NS | NS | NS | NS | NS | NS | NS | NS | NS |
| **What Do You Think Is the Most Effective Clinical-Pathological Communication Method?** | | | | | | | | | | | | | | | |
| Joint Slide Review for Difficult Cases | 2.48 | 1.25-4.90 | 0.009 | NS | NS | NS | NS | NS | NS | NS | NS | NS | NS | NS | NS |
| Pathologists Participating in Clinical Ward Rounds | NS | NS | NS | NS | NS | NS | 2.29 | 1.07-4.92 | 0.033 | NS | NS | NS | NS | NS | NS |
| Online Communication Platform | NS | NS | NS | NS | NS | NS | NS | NS | NS | NS | NS | NS | NS | NS | NS |
| Regular Clinical-Pathological Joint Training | 1.78 | 1.06-2.99 | 0.028 | NS | NS | NS | 2.05 | 1.03-4.08 | 0.04 | NS | NS | NS | NS | NS | NS |
| **Basic Situation of Primary Medical Institutions, Third-Party Institutions and AI** |  |  |  |  |  |  |  |  |  |  |  |  |  |  |  |
| **What do you think are the core needs of primary medical institutions for renal pathology?** | | | | | | | | | | | | | | | |
| Channels for rapid specimen transportation to external institutions | NS | NS | NS | NS | NS | NS | NS | NS | NS | NS | NS | NS | NS | NS | NS |
| Simplified pathology report | NS | NS | NS | 0.4 | 0.18-0.92 | 0.031 | NS | NS | NS | NS | NS | NS | NS | NS | NS |
| Telepathology consultation support | NS | NS | NS | NS | NS | NS | 2.93 | 1.26-6.82 | 0.013 | NS | NS | NS | NS | NS | NS |
| Renal pathology knowledge training tailored for primary institutions | 2.68 | 1.51-4.76 | 0.001 | NS | NS | NS | 2.84 | 1.27-6.37 | 0.011 | NS | NS | NS | NS | NS | NS |
| **What are the main restrictive factors for primary institutions to carry out renal pathology-related work?** | | | | | | | | | | | | | | | |
| Lack of specimen processing equipment | NS | NS | NS | NS | NS | NS | NS | NS | NS | NS | NS | NS | 1.97 | 1.05-3.70 | 0.034 |
| Insufficient technical personnel | NS | NS | NS | NS | NS | NS | 3.39 | 1.19-9.63 | 0.022 | NS | NS | NS | NS | NS | NS |
| High cost of external specimen transportation | NS | NS | NS | 0.37 | 0.17-0.85 | 0.019 | 2.41 | 1.16-5.01 | 0.018 | NS | NS | NS | NS | NS | NS |
| Unsmooth cooperation mechanism with pathology departments of superior hospitals | NS | NS | NS | NS | NS | NS | 2.82 | 1.37-5.81 | 0.005 | NS | NS | NS | NS | NS | NS |
| **Does your institution rely on third-party pathological testing institutions?** | | | | | | | | | | | | | | | |
| Yes (regularly send to professional pathological testing companies) | NS | NS | NS | NS | NS | NS | NS | NS | NS | NS | NS | NS | NS | NS | NS |
| Yes (regularly send to superior hospitals, e.g., primary hospitals send to tertiary hospitals) | NS | NS | NS | NS | NS | NS | NS | NS | NS | NS | NS | NS | 2.09 | 1.19-3.69 | 0.011 |
| No (with its own pathology department) | NS | NS | NS | NS | NS | NS | NS | NS | NS | NS | NS | NS | 0.33 | 0.16-0.66 | 0.007 |
| **What does your trust in third-party institutions mainly depend on?** | | | | | | | | | | | | | | | |
| Testing qualifications (e.g., certified laboratories) | NS | NS | NS | NS | NS | NS | NS | NS | NS | NS | NS | NS | NS | NS | NS |
| Timeliness of reports | NS | NS | NS | 0.29 | 0.12-0.73 | 0.008 | 3.35 | 1.49-7.51 | 0.003 | NS | NS | NS | NS | NS | NS |
| Ability to provide clinical interpretation suggestions | NS | NS | NS | 0.18 | 0.06-0.52 | 0.001 | 6.31 | 2.50-15.89 | <0.001 | NS | NS | NS | NS | NS | NS |
| Reasonableness of prices | NS | NS | NS | NS | NS | NS | 2.6 | 1.30-5.18 | 0.007 | NS | NS | NS | NS | NS | NS |
| **What do you think is the potential value of AI in renal pathology?** | | | | | | | | | | | | | | | |
| Rapid preliminary screening (e.g., identifying typical lesions, reducing manual workload) | NS | NS | NS | NS | NS | NS | NS | NS | NS | NS | NS | NS | NS | NS | NS |
| Quantitative analysis (e.g., automatic counting of glomerulosclerosis ratio) | NS | NS | NS | NS | NS | NS | NS | NS | NS | NS | NS | NS | NS | NS | NS |
| Assisting primary physicians in report interpretation (e.g., AI generates popularized conclusions) | NS | NS | NS | NS | NS | NS | 3.05 | 1.32-7.05 | 0.009 | NS | NS | NS | NS | NS | NS |
| No significant value (relying on pathologists' experience is more reliable) | NS | NS | NS | NS | NS | NS | NS | NS | NS | NS | NS | NS | NS | NS | NS |
| **What are your main concerns about the application of AI?** | | | | | | | | | | | | | | | |
| Misdiagnosis risk (especially for rare/complex cases) | NS | NS | NS | NS | NS | NS | NS | NS | NS | NS | NS | NS | NS | NS | NS |
| Data privacy leakage (patients' pathological images) | NS | NS | NS | NS | NS | NS | NS | NS | NS | NS | NS | NS | NS | NS | NS |
| Over-reliance on AI, weakening physicians' subjective judgment ability | 2.57 | 1.41-4.71 | 0.002 | NS | NS | NS | 10.62 | 3.57-31.61 | <0.001 | NS | NS | NS | NS | NS | NS |
| Lack of unified standards, inconsistent results among different AI systems | 1.80 | 1.06-3.04 | 0.028 | NS | NS | NS | NS | NS | NS | NS | NS | NS | NS | NS | NS |
| **ns, not significant;**  ❋The multivariate regression model was adjusted for the confounding effects of age, working years, professional title, and hospital grade.  ※ The multivariate regression model was adjusted for the confounding effects of sex, working years, professional title, and hospital grade.  † The multivariate regression model was adjusted for the confounding effects of sex, age, professional title, and hospital grade.  ƫ The multivariate regression model was adjusted for the confounding effects of sex, age, working years, and hospital grade.  § The multivariate regression model was adjusted for the confounding effects of sex, age, working years, and professional title. | | | | | | | | | | | | | | | |
