# Supplementary material for: Continuing medical education in renal pathology: current practices and needs among nephrologists
Source: BMC Med Educ. 2026 Feb 12;26:441. doi: 10.1186/s12909-026-08798-4 (PMC12997942; doi:10.1186/s12909-026-08798-4)
Supplement: Supplementary file 8 — Supplementary Material 8. [file 12909_2026_8798_MOESM8_ESM.docx]

**
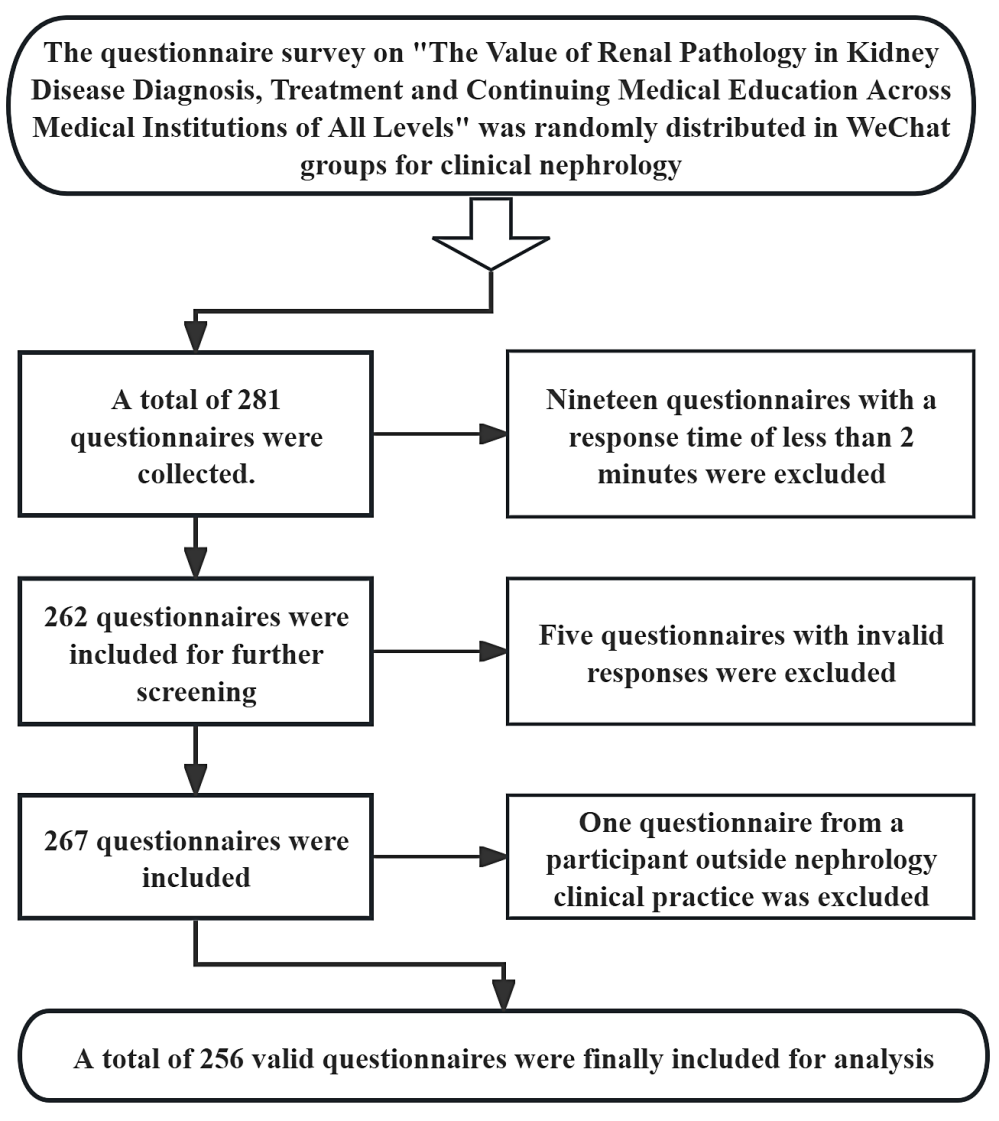
**

**Supplemental figure 1.** Flowchart of participant enrollment and exclusion

A total of 281 questionnaires were collected. After excluding 19 questionnaires with completion time <2 minutes, 5 invalid response questionnaires, and 1 questionnaire from non-nephrology related departments, 256 valid questionnaires were included in the final analysis. *Questionnaires completed in less than 2 minutes were discarded as hasty completion is suggestive of random and invalid responses, a determination based on the questionnaire’s pilot test.
